# Computer-Aided Discovery of EGFR G-Quadruplex Targeting Phenanthroimidazole Derivatives Inducing Concurrent Multi-Organelle Damage in Glioblastoma

**DOI:** 10.3390/antiox15080984

**Published:** 2026-08-08

**Authors:** Mengjiao She, Ao Yu, Xiaozhan Qiu, Wanyu Liu, Rui Chen, Renshan Deng, Huan Zeng, Chunling Zhu, Qiong Wu, Jinlan Meng, Wenjie Mei

**Affiliations:** 1 School of Pharmacy, Guangdong Engineering Technology Research Centre of Molecular Probe and Biomedicine Imaging, Guangdong Pharmaceutical University, Guangzhou 510006, China; 2112440268@stu.gdpu.edu.cn (M.S.); 2112542012@stu.gdpu.edu.cn (A.Y.); 2112440281@stu.gdpu.edu.cn (W.L.); 2112440279@stu.gdpu.edu.cn (R.C.); 2112440284@stu.gdpu.edu.cn (R.D.); 2112542175@stu.gdpu.edu.cn (H.Z.); 2112542188@stu.gdpu.edu.cn (C.Z.); 2 School of Basic Medical Sciences, Guangdong Pharmaceutical University, Guangzhou 510006, China; 2112340192@stu.gdpu.edu.cn; 3 Engineering Research Center of Small Molecule Drugs, Ministry of Education, Guangdong Pharmaceutical University, Guangzhou 510006, China; wuqiongniu.1113@163.com; 4 Department of Oncology, The Second Clinical Medial School of Gaungdong Pharmaceutical University, Guangdong Second Provincial General Hospital, Guangzhou 510006, China

**Keywords:** G-quadruplex DNA, phenanthroline-imidazole derivatives, Glioblastoma multiforme, oxidative stress

## Abstract

The urgent need for novel glioblastoma (GBM) therapies has motivated the exploration of EGFR G-quadruplex (G4) targeting, which suppresses oncogene transcription independently of kinase activity. However, existing EGFR G4 ligands lack subtype selectivity. Through computer-aided design and systematic bioisosteric modifications of the 1*H*-imidazo[4,5-f][1,10]phenanthroline scaffold, we synthesized a series of derivatives. We evaluated their G4 interactions by molecular docking, UV-Vis, FRET, ITC, and CD. Compound **3**, bearing a 6-bromopiperonyl group, exhibited nanomolar affinity for EGFR G4 (Kd = 102 nM) with ~146-fold selectivity over K-Ras G4, stabilizing it via end-stacking. It potently inhibited U87-MG glioblastoma cell proliferation (IC_50_ = 0.49 μM), with a wide safety margin relative to normal HMC3 microglia (safety index = 14.18). Mechanistically, compound **3** triggered DNA damage, mitochondrial oxidative stress, and sequential multi-organelle damage to mitochondria, lysosomes, and the endoplasmic reticulum, while impairing cell migration and invasion. These findings establish compound **3** as a uniquely selective EGFR G4 stabilizer, offering a promising multi-organelle-damage strategy for glioblastoma therapy.

## 1. Introduction

Glioblastoma multiforme (GBM) is the most common and aggressive primary brain tumor in adults. Despite standard surgery, temozolomide, and radiotherapy, median survival is only ~15 months, and the 5-year survival rate is below 10% [1]. Most molecularly targeted agents, including EGFR tyrosine kinase inhibitors, have failed to improve survival in randomized trials, largely due to limited penetration across the blood–brain barrier, profound molecular heterogeneity, compensatory signaling, and intrinsic resistance in glioma stem cells [2,3]. EGFR amplification occurs in approximately 57% of GBM cases. Nevertheless, kinase inhibitors yield only transient responses due to downstream network complexity and bypass mechanisms, underscoring the need for non-kinase-dependent EGFR strategies [4]. Given the limited clinical benefit of EGFR kinase inhibitors in glioblastoma, targeting the G-quadruplex in the EGFR promoter to suppress gene expression at the transcriptional level may circumvent the limitations of conventional inhibitors; however, existing ligands lack subtype selectivity, creating an urgent need for highly selective stabilizers. To this end, this study adopted the core scaffold of the clinical-stage phenanthroimidazole compound APTO-253 as a starting point. It employed computer-aided drug design to guide systematic bioisosteric modifications, aiming to identify novel lead compounds with high affinity and subtype selectivity for EGFR G4.

G-quadruplex (G4) structures, formed by guanine-rich sequences, have emerged as anticancer targets owing to their roles in oncogene transcription [5]. The EGFR promoter contains G4-forming sequences, providing a structural basis for direct transcriptional repression of EGFR [6]. Stabilizing EGFR G4 with small molecules can block EGFR expression independently of kinase activity, thereby potentially circumventing resistance from bypass signaling [7]. Classical EGFR TKIs inhibit phosphorylation by competing for ATP but are limited by poor penetration of the blood–brain barrier and kinase-domain mutations. Conversely, targeting the EGFR promoter G-quadruplex suppresses transcription independently of kinase activity, thus circumventing these limitations. Several EGFR G4 ligands have been reported, including the quinoline CK-14 and the naphthyridine Naph-1, both of which suppress EGFR transcription and glioma cell proliferation [8,9]. However, the phenanthroline-imidazole APTO-253 stabilizes c-Myc and K-Ras G4s but exhibits weak affinity for EGFR G4s, highlighting a persistent binding selectivity gap [10]. Most current G4 ligands lack sufficient subtype selectivity and discriminate poorly between duplex DNA and other nucleic acid structures, leading to off-target effects that limit clinical translation [11,12]. Designing highly selective EGFR G4 stabilizers based on EGFR’s unique structural features, therefore, represents a promising approach to developing precision therapies for GBM [6].

1*H*-Imidazo[4,5-f][1,10]phenanthroline provides an extended π-plane for G-quartet stacking, an imidazole NH hydrogen-bond donor, and a modifiable C2 aryl position amenable to bioisosterism and introduction of hydrogen- and halogen-bond donors/acceptors. APTO-253 stabilizes multiple promoter G4s, and previous derivatives have achieved selectivity toward VEGF, Bcl-2, and c-Kit G4s, demonstrating that fine-tuning the C2 substituent can direct selectivity [10,13,14,15]. Despite this potential, highly selective ligands for GBM-relevant targets, especially EGFR G4, remain absent. Computer-aided drug design (CADD), integrating molecular docking, dynamics simulations, and density functional theory calculations, can efficiently guide structural optimization to enhance G4 topology selectivity, offering a powerful approach for developing novel EGFR G4-selective stabilizers [16].

**Scheme 1:**
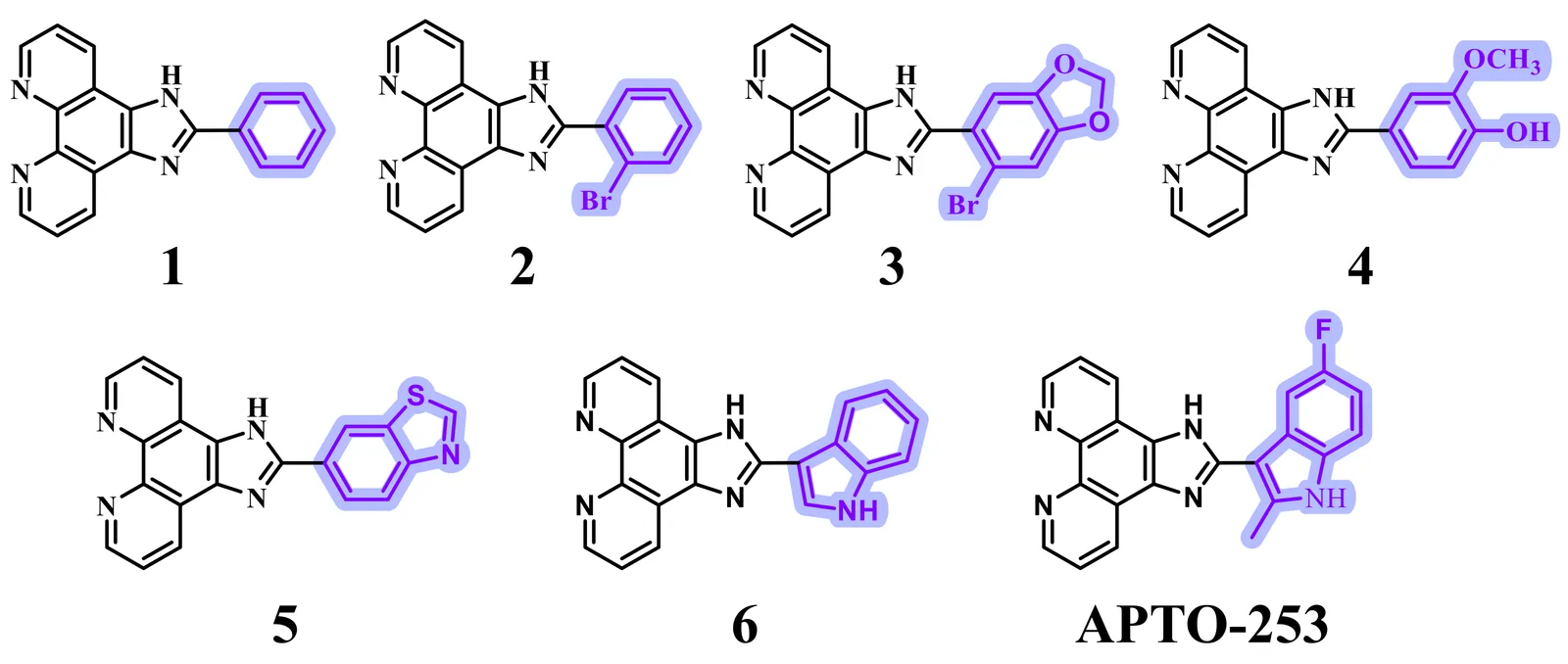
The molecular structures of the designed G4 DNA stabilizing ligands **1**–**6** and the classical G-quadruplex DNA binder APTO-253.

In this study, using APTO-253 as a lead scaffold and a CADD-driven strategy, we synthesized a series of phenanthroline-imidazole derivatives (**1**–**6**) with systematic C2 bioisosteric modifications (Scheme 1). Molecular docking identified compound **3**, bearing a 6-bromopiperonyl group, as a highly selective EGFR G4 binder with nanomolar affinity (Kd = 102 nM) and ~146-fold selectivity over K-Ras G4. Biophysical analyses indicated an end-stacking stabilization mode. Compound **3** potently inhibited U87-MG glioblastoma cell proliferation (IC_50_ = 0.49 μM) with a wide safety margin relative to normal HMC3 microglia (safety index 14.18). Mechanistically, it induced DNA damage, mitochondrial oxidative stress, and concurrent multi-organelle damage to mitochondria, lysosomes, and the endoplasmic reticulum, while also suppressing cell migration and invasion. These findings establish compound **3** as a promising EGFR G4-targeted lead and provide new insights into the antitumor mechanism of G4 ligands through synergistic multi-organelle damage.

## 2. Materials and Methods

### 2.1. Chemicals and Materials

All chemicals and solvents were purchased from Aladdin and Maclean’s and were used without further purification unless otherwise noted. Solvents were dried and purified following standard procedures before use. Microwave-assisted synthesis of the phenanthroline-imidazole derivatives was carried out on an Anton Paar Monowave 300 reactor (Anton Paar GmbH, Graz, Austria). Mass spectra were acquired on an Agilent 1100 ESI-MS system (Agilent Technologies, Santa Clara, CA, USA) with ethanol as the solvent. NMR spectra were recorded on Bruker DRX 500 (Bruker Biospin, Rheinstetten, Germany) or 600 MHz spectrometers (Bruker Biospin, Rheinstetten, Germany) using DMSO-*d*_6_ as the solvent; chemical shifts are reported in ppm relative to tetramethylsilane. All six synthesized phenanthroimidazole derivatives were obtained as yellow solid powders.

### 2.2. Synthesis and Characterization

#### 2.2.1. 2-Phenyl-1*H*-imidazo[4,5-f][1,10]phenanthroline (1)

A general procedure was used to prepare all target compounds. A mixture of 1,10-phenanthroline-5,6-dione (100.0 mg, 0.476 mmol), the corresponding benzaldehyde (75.8 mg, 0.714 mmol), and ammonium acetate (5 g) in glacial acetic acid (15 mL) was stirred at room temperature for 10 min. The resulting solution was then transferred into a sealed 30 mL quartz tube and subjected to microwave irradiation in a Monowave 300 reactor at 100 °C for 30 min. After cooling to room temperature, the reaction mixture was diluted with distilled water (30 mL) and adjusted to pH 7.0 with concentrated aqueous ammonia. The resulting precipitate was collected by filtration, and the crude product was purified by column chromatography on silica gel using absolute ethanol as the eluent, affording 75%; melting point 314.83 ± 0.63 °C. ESI-MS (EtOH) *m*/*z* calculated for C_19_H_13_N_4_ [M+H]^+^ 297.1135, found 297.1125. ^1^H NMR (600 MHz, DMSO-*d*_6_) δ 9.03 (dd, *J* = 4.3, 1.7 Hz, 2H), 8.93 (dd, *J* = 8.1, 1.8 Hz, 2H), 8.32–8.27 (m, 2H), 7.83 (dd, *J* = 8.1, 4.3 Hz, 2H), 7.61 (dd, *J* = 8.4, 7.1 Hz, 2H), 7.54–7.50 (m, 1H). ^13^C NMR (151 MHz, DMSO-*d*_6_) δ 151.12, 148.26, 144.04, 130.56, 130.11, 130.06, 129.48, 126.71, 123.76.

#### 2.2.2. 2-(2-Bromophenyl)-1*H*-imidazo[4,5-f][1,10]phenanthroline (2)

Compound **2** was synthesized following the general procedure described above, using 2-bromobenzaldehyde (132.0 mg, 0.714 mmol) in place of benzaldehyde, affording 66%; melting point 304.87 ± 0.71 °C. ESI-MS (EtOH) *m*/*z* calculated for C_19_H_12_BrN_4_ [M+H]^+^ 375.0240, found 375.0245. ^1^H NMR (500 MHz, DMSO-*d*_6_) δ 13.92 (s, 1H), 9.06 (dd, *J* = 4.3, 1.9 Hz, 2H), 8.88 (d, *J* = 8.1 Hz, 2H), 7.90 (d, *J* = 8.1 Hz, 1H), 7.88–7.83 (m, 3H), 7.65–7.61 (m, 1H), 7.54 (td, *J* = 7.7, 1.9 Hz, 1H). ^13^C NMR (126 MHz, DMSO-*d*_6_) δ 150.08, 148.45, 144.11, 133.83, 132.88, 132.81, 132.03, 130.05, 128.38, 123.87, 122.54.

#### 2.2.3. 2-(6-Bromobenzo[d][1,3]dioxol-5-yl)-1*H*-imidazo[4,5-f][1,10]phenanthroline (3)

Compound **3** was synthesized following the general procedure described above, using 6-bromobenzo[d][1,3]dioxole-5-carbaldehyde (163.5 mg, 0.714 mmol) in place of benzaldehyde, affording 72%; melting point 320.17 ± 0.45 °C. ESI-MS (EtOH) *m*/*z* calculated for C_20_H_12_BrN_4_O_2_ [M+H]^+^ 419.01, found 419.10. ^1^H NMR (600 MHz, DMSO-*d*_6_) δ 13.81 (s, 1H), 9.05 (t, *J* = 3.4 Hz, 2H), 8.88 (d, *J* = 8.2 Hz, 1H), 8.85 (d, *J* = 8.1 Hz, 1H), 7.84 (td, *J* = 8.0, 3.7 Hz, 2H), 7.48 (s, 1H), 7.42 (d, *J* = 4.5 Hz, 1H), 6.23 (s, 2H). ^13^C NMR (151 MHz, DMSO-*d*_6_) δ 152.87, 148.94, 148.81, 147.30, 146.67, 142.90, 134.44, 128.99, 125.20, 124.81, 122.90, 122.66, 118.68, 112.87, 112.68, 112.43, 110.91, 106.84, 102.71, 102.07.

#### 2.2.4. 4-(1*H*-Imidazo[4,5-f][1,10]phenanthrolin-2-yl)-2-methoxyphenol (4)

Compound **4** was synthesized following the general procedure described above, using 4-hydroxy-3-methoxybenzaldehyde (108.6 mg, 0.714 mmol) in place of benzaldehyde, affording 63%; melting point 274.90 ± 0.85 °C. ESI-MS (EtOH) *m*/*z* calculated for C_20_H_14_N_4_O_2_ [M+Na]^+^ 365.0998, found 365.1010.^1^H NMR (500 MHz, DMSO-*d*_6_) δ 9.02 (dd, *J* = 4.3, 1.7 Hz, 2H), 8.92–8.90 (m, 2H), 8.28 (d, *J* = 8.2 Hz, 2H), 7.82 (dd, *J* = 7.8, 4.2 Hz, 2H), 7.59 (d, *J* = 8.2 Hz, 3H), 5.17 (s, 3H). ^13^C NMR (126 MHz, DMSO-*d*_6_) δ 170.32, 150.17, 147.86, 143.61, 137.60, 129.68, 129.63, 128.53, 128.04, 126.26, 123.31, 65.11.

#### 2.2.5. 6-(1*H*-Imidazo[4,5-f][1,10]phenanthrolin-2-yl)benzo[d]thiazole (5)

Compound **5** was synthesized following the general procedure described above, using benzo[d]thiazole-6-carbaldehyde (116.5 mg, 0.714 mmol) in place of benzaldehyde, affording 69%; melting point 273.20 ± 0.29 °C. ESI-MS (EtOH) *m*/*z* calculated for C_20_H_12_N_5_S [M+H]^+^ 354.0808, found 354.0807. ^1^H NMR (600 MHz, DMSO-*d*_6_) δ 9.53–9.49 (m, 2H), 9.27–9.25 (m, 1H), 9.15 (dt, *J* = 4.7, 2.3 Hz, 2H), 9.12 (t, *J* = 2.1 Hz, 1H), 8.59–8.55 (m, 1H), 8.26 (dd, *J* = 8.6, 4.5 Hz, 1H), 8.14 (dt, *J* = 8.3, 3.9 Hz, 2H). ^13^C NMR (151 MHz, DMSO-*d*_6_) δ 172.15, 155.96, 153.68, 151.88, 144.52, 140.80, 136.13, 134.51, 134.13, 124.52, 123.16, 120.31.

#### 2.2.6. 2-(1*H*-Indol-3-yl)-1H-imidazo[4,5-f][1,10]phenanthroline (6)

Compound **6** was synthesized following the general procedure described above, using 1*H*-indole-3-carbaldehyde (103.6 mg, 0.714 mmol) in place of benzaldehyde, affording 71%; melting point 311.80 ± 1.43 °C. ESI-MS (EtOH) *m*/*z* calculated for C_21_H_14_N_5_ [M+H]^+^ 336.1244, found 336.1242. ^1^H NMR (500 MHz, DMSO-*d*_6_) δ 13.43 (s, 1H), 11.76 (s, 1H), 9.11–8.98 (m, 3H), 8.92–8.68 (m, 2H), 8.29–8.22 (m, 1H), 7.85 (dd, *J* = 8.2, 4.3 Hz, 2H), 7.61–7.53 (m, 1H), 7.31–7.24 (m, 2H). ^13^C NMR (126 MHz, DMSO-*d*_6_) δ 149.38, 147.80, 143.75, 137.00, 130.10, 129.74, 126.04, 125.66, 125.35, 124.24, 123.73, 122.82, 122.02, 120.79, 119.64, 112.46, 107.13.

### 2.3. Molecular Docking

Geometries of phenanthroline-imidazole derivatives were optimized using ADF2019.104 at the GGA: BP86 level with Mopac, and initial coordinates were generated with Mercury. The docking protocol was validated by re-docking the co-crystallized ligand into its original binding pocket using AutoDock4.2 and calculating the RMSD between the docked pose and the experimental conformation. For the EGFR G4 structure (8JFQ), the re-docked pose yielded an RMSD of 1.82 Å, indicating that the docking parameters can accurately reproduce the experimental binding mode. Docking against G4 DNA structures (c-Myc [2L7V], K-Ras [5I2V], VEGF [2M27], Bcl-2 [2F8U], Tel26 [2MBJ], c-Kit1 [3GXR], c-Kit2 [2KYO], EGFR [8JFQ]) and dsDNA (5TGG) was performed with AutoDock4.2 using the Lamarckian genetic algorithm. The grid box (126 × 126 × 126 points) was centered at (8.124, −7.145, 11.327). The best binding conformation was selected from ten independent runs based on the largest cluster and lowest energy [11].

### 2.4. Molecular Dynamics Simulations

Docked complexes served as initial structures for MD simulations with GROMACS 2019.5_GPU using Amber force fields. Small-molecule parameters were assigned via the ATB server, and G4 DNA charges were described with Amber 99SB-ILDN. K^+^ ions and water molecules were added to mimic physiological conditions, and a Langevin thermostat was used to control the temperature. RMSD analysis was performed using Ptraj, and visualization was performed using PyMOL (pymol-3.2.0a0-cp314-cp314-win_amd64), VMD (vmd 1.9.3), and QtGrace (QtGrace 2.6) [6].

### 2.5. Spectroscopic Studies of DNA Binding

EGFR, K-Ras, and dsDNA oligonucleotides (General Biosystems) were prepared in 10 mM Tris-HCl-KCl buffer, heated to 95 °C for 5 min, and slowly cooled. UV-Vis absorption spectra of compound **3** (20 μM) were recorded on a Shimadzu UV-2550 spectrophotometer (Shimadzu Corporation, Kyoto, Japan) with increasing DNA concentrations; fluorescence was measured on an RF-5301 fluorospectrophotometer (Shimadzu Corporation, Kyoto, Japan). Binding constants were derived using the Stern-Volmer equation. FRET melting assays were carried out on a Bio-Rad iQ5 system (Bio-Rad Laboratories, Inc., Hercules, CA, USA) with labeled oligonucleotides (1.0 μM) in the absence and presence of compound **3** (2.0, 4.0 μM), and selectivity was evaluated by competition with ds26 duplex DNA (0.2 μM). Isothermal titration calorimetry (Nano ITC, Microcal, TA Instruments, New Castle, DE, USA) was performed at 25 °C by injecting 2 μL aliquots of compound **3** (10 μM) into G4 DNA (10 μM) with 120 s spacing and 350 rpm stirring; binding parameters were obtained using Origin 2024. CD spectra were collected on a Jasco J-815 spectropolarimeter (JASCO Corp., Tokyo, Japan) with a 10 mm cuvette; the compound was titrated into 2.0 μM G4 DNA in 10 mM Tris-HCl and 0.1 M KCl/NaCl (pH 7.4), incubated for 15 min, and scanned at 50 nm/min [10].

### 2.6. Lipophilicity (Log P) and pKa

Log Po/w was measured by the shake-flask method with mutually saturated 1-octanol and water after 24 h equilibration (25 °C, 150 rpm). Compound concentrations in both phases were quantified by UV-Vis (Shimadzu 2550, λ = 277 nm) using validated calibration curves. pKa values were determined spectrophotometrically at room temperature with a Sartorius PB-10 pH meter (Sartorius AG, Göttingen, Germany); spectral changes in acid/base solutions were recorded, and pKa was calculated from absorbances of the fully acidic, fully basic, and mixed forms [14].

### 2.7. MTT Assay

U87-MG (human glioblastoma), LN229 (human glioblastoma), MCF-7 (human breast cancer), and A549 (human non-small cell lung adenocarcinoma) cells were obtained from the China Center for Type Culture Collection (CCTCC), and HMC3 (human normal microglia) cells were obtained from the Tenth People’s Hospital of Tongji University. These cells were seeded in 96-well plates (5 × 10^3^/well, *n* = 3) and cultured for 24 h. Compounds were applied at graded concentrations for 72 h, followed by MTT (5 mg/mL, 20 μL) for 4 h. Formazan was dissolved in DMSO, and absorbance at 570 nm was read on a Thermo Multiskan GO (Thermo Scientific, Vantaa, Finland). IC_50_ values were calculated with GraphPad Prism 8.02.

### 2.8. EdU Proliferation Assay

U87-MG cells (5 × 10^4^/well, 6-well plates) were treated with compound **3** (0–1 μM) for 72 h, incubated with 10 μM EdU for 2 h, fixed, permeabilized, and stained using the BeyoClick^TM^ EdU Kit (Beyotime Biotechnology, Shanghai, China). Nuclei were counterstained with DAPI. Images were captured on a Leica DMI8 (Leica Microsystems, Wetzlar, Germany), and the EdU-positive rate was determined as EdU^+^/DAPI^+^ × 100% [10].

### 2.9. Bio-TEM

U87-MG cells (5 × 10^5^/dish) were treated with compound **3** (0, 1 μM) for 72 h, fixed with 2.5% glutaraldehyde, dehydrated in ethanol/acetone, embedded in epoxy resin, and sectioned (70 nm). Ultrathin sections on 200-mesh grids were stained with uranyl acetate and lead citrate and imaged on an FEI Tecnai G2 Spirit (Thermo Scientific, Waltham, MA, USA) at 120 kV [10,14].

### 2.10. Cell Migration and Invasion

Wound healing: confluent U87-MG cells were scratched, washed, and incubated with 0.5 μM compound **3**; time-lapse imaging was performed over 12 h. Gelatin degradation: FITC-gelatin-coated dishes were crosslinked with glutaraldehyde, activated with NaBH_4_, and seeded with U87-MG cells (5 × 10^4^/mL) mixed with compound **3** (0, 0.0625, 0.125 μM). After 24 h, cells were fixed, permeabilized, stained with rhodamine-phalloidin, and imaged on a Zeiss LSM980 (Carl Zeiss Microscopy GmbH, Jena, Germany) [14].

### 2.11. Flow Cytometry

U87-MG cells (1 × 10^5^/well) were treated with compound **3** (0–1 μM) for 72 h. For the cell cycle, cells were fixed in 70% ethanol and stained with PI/RNase. Apoptosis was assessed with FITC-Annexin V/PI, mitochondrial membrane potential with JC-1, and mtROS with MitoSOX. Samples were analyzed on a Beckman Coulter Epics XL-MCL (Beckman Coulter, Brea, CA, USA) [10].

### 2.12. Cellular Redox Status

MDA was measured with the Lipid Peroxidation MDA Assay Kit (Beyotime) at 532 nm. GSH/GSSG ratios were determined using the GSH/GSSG Assay Kit after deproteinization. ATP was quantified with the ATP Assay Kit (Beyotime) from RIPA lysates. NAD^+^/NADH was extracted by acid/base methods and measured with the NAD^+^/NADH Assay Kit (Beyotime) at 450 nm [10].

### 2.13. Calcium and Organelle Staining

U87-MG cells on coverslips were treated with compound **3** for 72 h, then stained with Fluo-2-AM, MitoTracker Green, Hoechst 33342, and ER-Tracker (all 1:500) for 30 min at 37 °C. Fluorescent images were acquired on a Leica DMI8 [17].

### 2.14. Immunofluorescence

Treated cells were fixed, permeabilized, blocked with 2% BSA, and incubated with primary antibodies overnight at 4 °C, followed by secondary antibodies. Nuclei were stained with DAPI. Images were captured on a Leica DMI8 [10].

### 2.15. Western Blotting

U87-MG cells treated with compound **3** (0–1 μM, 72 h) were lysed in RIPA buffer, and protein concentrations were determined by Bradford assay. Equal amounts of protein were resolved by 10% SDS-PAGE, transferred to nitrocellulose, blocked, and probed with primary (1:1000) and secondary (1:2000) antibodies. Bands were visualized on a LI-COR Odyssey CLx (LI-COR Biosciences, Lincoln, NE, USA) [10].

### 2.16. Zebrafish Glioblastoma Model

DiI-labeled U87-MG cells (1 × 10^7^/mL) were stereotactically implanted into the diencephalic ventricle of 48 hpf fli1a: EGFP embryos. Engrafted embryos (*n* = 6/group) were maintained in 0.002% PTU and exposed to compound **3** (0, 5, 10, 20 μM) with daily water changes. Fluorescent images were taken every 24 h on a Leica DMI8. Tumor fluorescence was quantified with ImageJ 1.52a (Wayne Rasband, National Institutes of Health, USA) and analyzed with GraphPad Prism. All procedures complied with relevant ethical regulations [10,14].

### 2.17. Statistical Analysis Section

All experiments were performed with at least three technical replicates, and data are presented as the mean ± standard error of the mean (SEM). Technical replicates are multiple independent measurements of the same sample under identical experimental conditions, used to assess the precision of the detection method and operational reproducibility. Statistical analyses were conducted using GraphPad Prism 8.02 software. Group differences were compared using one-way analysis of variance (ANOVA) followed by appropriate post hoc tests, with significance levels set at * *p* < 0.05, ** *p* < 0.01, and *** *p* < 0.001.

## 3. Results

### 3.1. Molecular Design and Synthesis

Based on the 1*H*-imidazo[4,5-f][1,10]phenanthroline core of APTO-253 as a privileged scaffold, we designed six derivatives with systematic modifications at the 2-aryl position. The phenanthroline moiety provides an extended π-plane for G-quartet stacking, while the imidazole NH serves as a hydrogen-bond donor. To optimize non-covalent interactions with different G4 DNA, we applied bioisosterism, π-system extension, and the introduction of hydrogen- and halogen-bond donors/acceptors [18,19]. Compound **1** bears an unsubstituted phenyl to evaluate hydrophobic stacking independently of fluorine effects. Compound **2** incorporates an ortho-bromine to introduce a halogen-bond donor and modulate the interplanar dihedral angle. Compound **3** features a 5-bromo-1,3-benzodioxole (6-bromopiperonyl) group, where the methylenedioxy moiety provides hydrogen-bond acceptors that cooperate with bromine-mediated halogen bonding to enhance G4 selectivity. Compound **4** contains a 3-methoxy-4-hydroxyphenyl fragment that forms specific hydrogen-bond networks via the phenolic hydroxyl and methoxy groups. Compound **5** replaces the phenyl with a benzo[d]thiazol-2-yl group, extending the π-system and increasing polarizability through the thiazole sulfur and nitrogen atoms. Compound **6** introduces an indol-2-yl group, expanding the conjugation plane and offering an additional hydrogen-bond donor through the indole NH for directional interactions with guanine carbonyls or the phosphodiester backbone (Scheme 1). This focused library of C2-modified derivatives, encompassing diverse electronic, steric, and hydrogen- and halogen-bonding profiles, was thus constructed to elucidate the key structural determinants governing G4 binding affinity and selectivity.

### 3.2. Single-Crystal Structural Analysis

**Figure 1:**
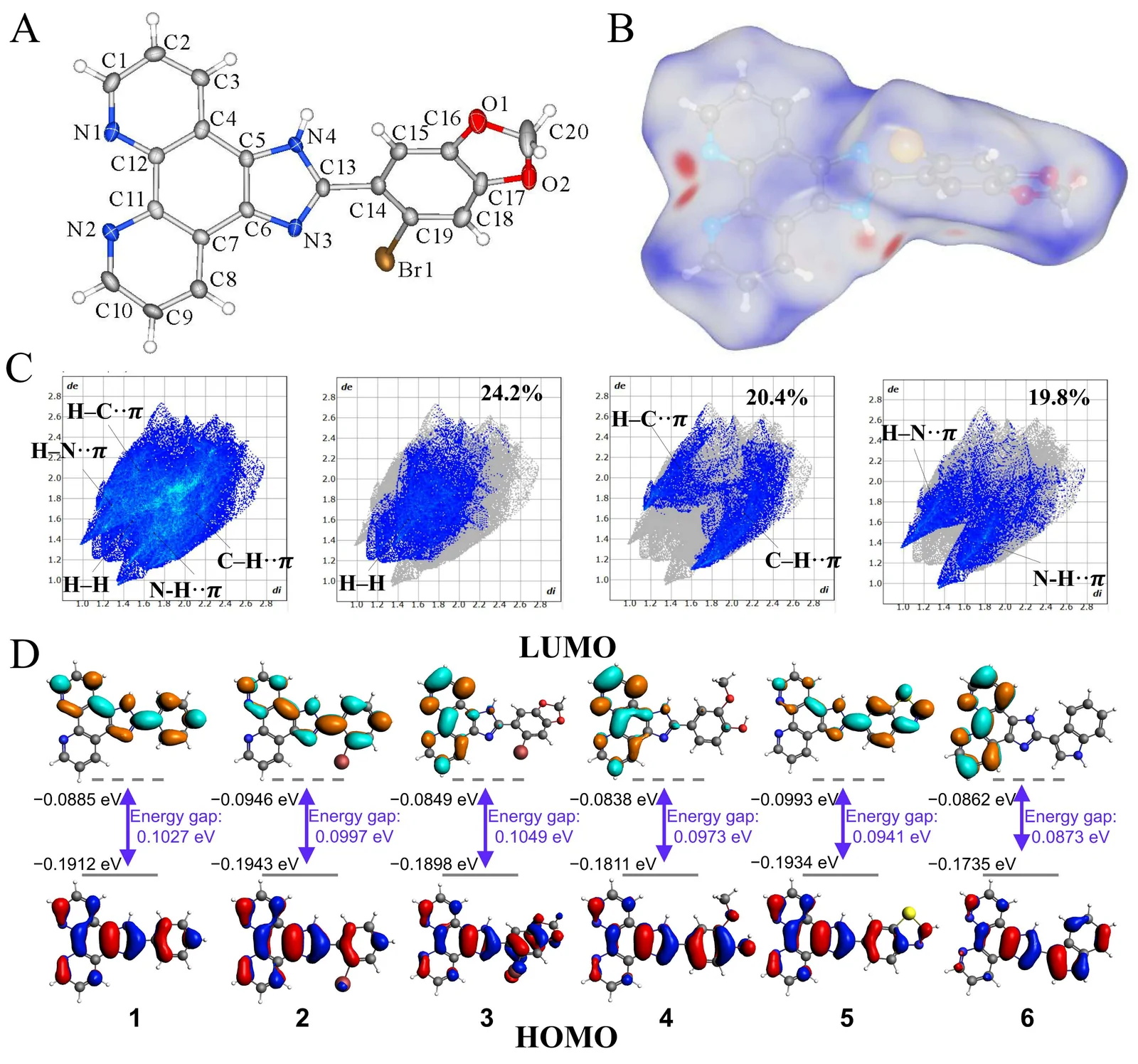
(**A**) Single-crystal X-ray structure of compound **3**. (**B**) Hirshfeld surface analysis of compound **3**, where the color gradient from blue to red denotes regions of increasing intermolecular interaction strength. (**C**) Two-dimensional (2D) fingerprint plots of compound **3**, displaying the full profile and the resolved C–H, H–H, and N–H contacts, along with their respective percentage contributions to the total Hirshfeld surface area. (**D**) HOMO–LUMO energy gaps (in eV) of compounds **1**–**6**, calculated at the DFT level.

To establish the 3D conformational basis for selective G4 DNA recognition by compound **3**, we first determined its single-crystal structure. The dihedral angle between the phenanthroline-imidazole core and the 6-bromo-1,3-benzodioxole plane is 51.19°, producing a rigid V-shaped non-coplanar conformation (Figure 1A). This twisted geometry avoids face-to-face π–π stacking, instead promoting edge-to-face and heteroatom–π interactions as the dominant assembly driving forces. The imidazole N–H and aromatic C–H groups project toward adjacent π-planes, while the dioxole oxygen atoms serve as lateral anchors via C–H···O and lone-pair···π contacts, and the bromine atom provides directional nodes through σ-hole-driven C–Br···π halogen bonds. This preorganized V-shaped architecture suppresses self-aggregation and orients the functional groups for specific directional interactions, laying the structural foundation for selective G4 binding [20].

To quantify the intermolecular interactions in the crystal lattice, we performed Hirshfeld surface analysis (Figure 1B,C). H···H van der Waals contacts contribute only 24.2% of the total surface, whereas X–H···π directional interactions collectively account for 58.9%, with C–H···π, N–H···π, O–H···π, and Br–H···π representing 20.4%, 19.8%, 10.6%, and 8.1%, respectively. This distribution demonstrates that edge-to-face and heteroatom–π recognition dominate crystal packing rather than nonspecific hydrophobic forces. The collective effect of these weak interactions forms a non-planar, folded interlocking lamellar network that mirrors the directional requirements for recognizing the guanine-rich G4 surface, highlighting a recognition surface enriched with geometrically complementary weak-bond sites essential for selective G4 recognition [21].

### 3.3. Electronic Properties Analysis by DFT Calculation

Having elucidated the conformational and interaction profiles, we employed DFT calculations to evaluate the electronic properties underpinning G4 binding (Figure 1D). The HOMO–LUMO gaps across the series range from 0.0873 to 0.1049 eV, with compound **3** exhibiting a gap of 0.1049 eV. These narrow gaps and pronounced π-electron delocalization across the fused scaffold confer strong polarizability and charge-transfer ability, promoting π–π stacking and electrostatic complementarity during G4 association. The minimal gap variation across derivatives (Δeg = 0.0176 eV) indicates that the rigid heteroaromatic core governs the electronic structure, with terminal substituents exerting only marginal influence on energy-level alignment. This electronic robustness underscores the scaffold’s excellent modifiability, allowing the introduction of diverse recognition motifs at the C2 position to optimize selectivity and pharmacokinetic profiles without perturbing the core pharmacophore or its binding mode, thereby reducing off-target risks. Together with the structural and interaction analyses, these electronic features suggested that compound **3** combines a preorganized rigid conformation, a tunable multisite recognition surface, and favorable frontier orbital properties, providing a comprehensive structural basis for its efficient and highly selective stabilization of EGFR G4 DNA [22].

### 3.4. CADD-Driven Screening for G-Quadruplex DNA Binders

**Figure 2:**
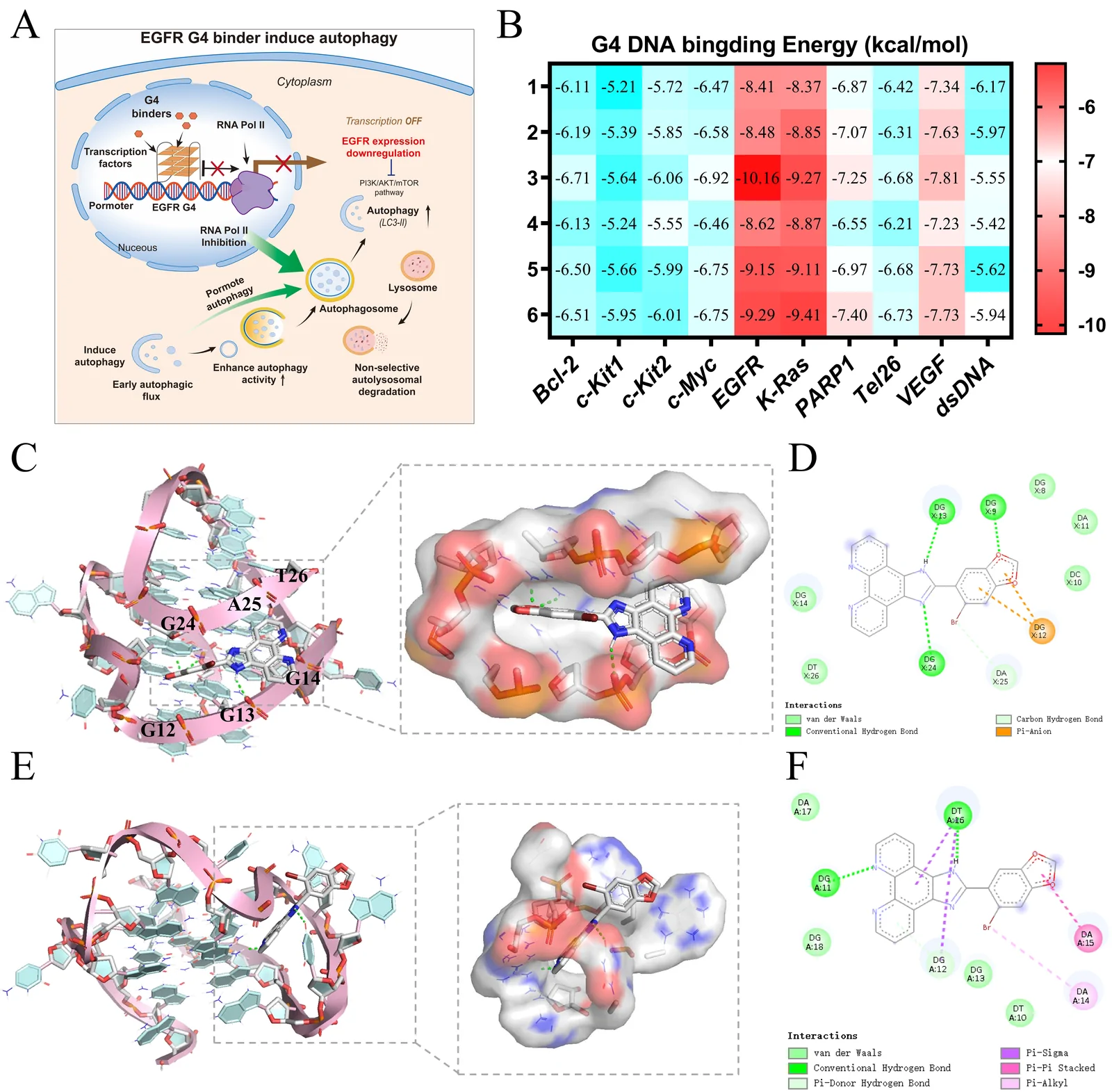
Binding properties and mechanistic insights into compound **3** targeting G-quadruplex DNAs. (**A**) Schematic illustration of the proposed molecular mechanism by which EGFR G4 DNA ligands stabilize G-quadruplexes, leading to suppressed EGFR transcription, induced autophagy, and the eventual inhibition of cancer cell proliferation. (**B**) Binding energy heatmap depicting the docking scores of compounds **1**–**6** against Bcl-2, c-Kit1, c-Kit2, c-Myc, EGFR, K-Ras, PARP1, Tel26, VEGF G-quadruplexes (G4s), and double-stranded DNA (dsDNA). (**C**,**D**) Docking pose and two-dimensional (2D) interaction diagram of compound **3** bound to the EGFR G4 DNA. (**E**,**F**) Docking pose and 2D interaction diagram of compound **3** bound to the K-Ras G4 DNA.

Molecular docking revealed that APTO-253 exhibits moderate affinity toward several G4 structures, with the strongest binding to K-Ras (−7.92 kcal/mol) and appreciable affinity for Bcl-2, PARP1, Tel26, and c-Kit2 (−7.19 to −7.26 kcal/mol), but weak binding to EGFR and VEGF (−6.15 and −5.63 kcal/mol) and no selectivity for VEGF over dsDNA [10]. Upon introducing diverse C2-aryl substituents, the six derivatives displayed markedly altered selectivity profiles (Figure 2A), with substantially enhanced affinity for EGFR and K-Ras G4 and diminished binding to Bcl-2, c-Kit-1, and c-Kit-2. All new compounds maintained low dsDNA affinity (−5.42 to −6.17 kcal/mol), yielding pronounced selectivity windows. Compound **3**, bearing a 6-bromopiperonyl group, exhibited the most favorable profile, with binding energies of −10.16 kcal/mol for EGFR G4 and −9.27 kcal/mol for K-Ras G4, and a dsDNA affinity of only −5.55 kcal/mol, corresponding to selectivity indices of 4.61 and 3.72 kcal/mol, respectively, far surpassing those of APTO-253 (0.52 and 2.29 kcal/mol) [10]. Compounds **4**, **5**, and **6** also significantly improved EGFR/K-Ras recognition with selectivity indices in the 3.2–3.5 kcal/mol range, whereas compounds **1** and **2** showed more modest improvements accompanied by reduced affinity for certain G4 subtypes. Structure–activity analysis indicated that extending the π-system with electron-rich benzodioxole, benzothiazole, or indole moieties, combined with halogen or hydrogen bonding from bromine or phenolic/methoxy groups, strongly enhances stacking and polar interactions within the EGFR and K-Ras G4 binding pockets. However, this strategy is not universally applicable to structurally distinct G4s such as Bcl-2 and c-Kit. Overall, precise bioisosteric and functional-group tuning at the 2-aryl position effectively reshapes the G4-subtype selectivity spectrum, providing a rational basis for the design of highly selective EGFR G4 ligands.

**Figure 3:**
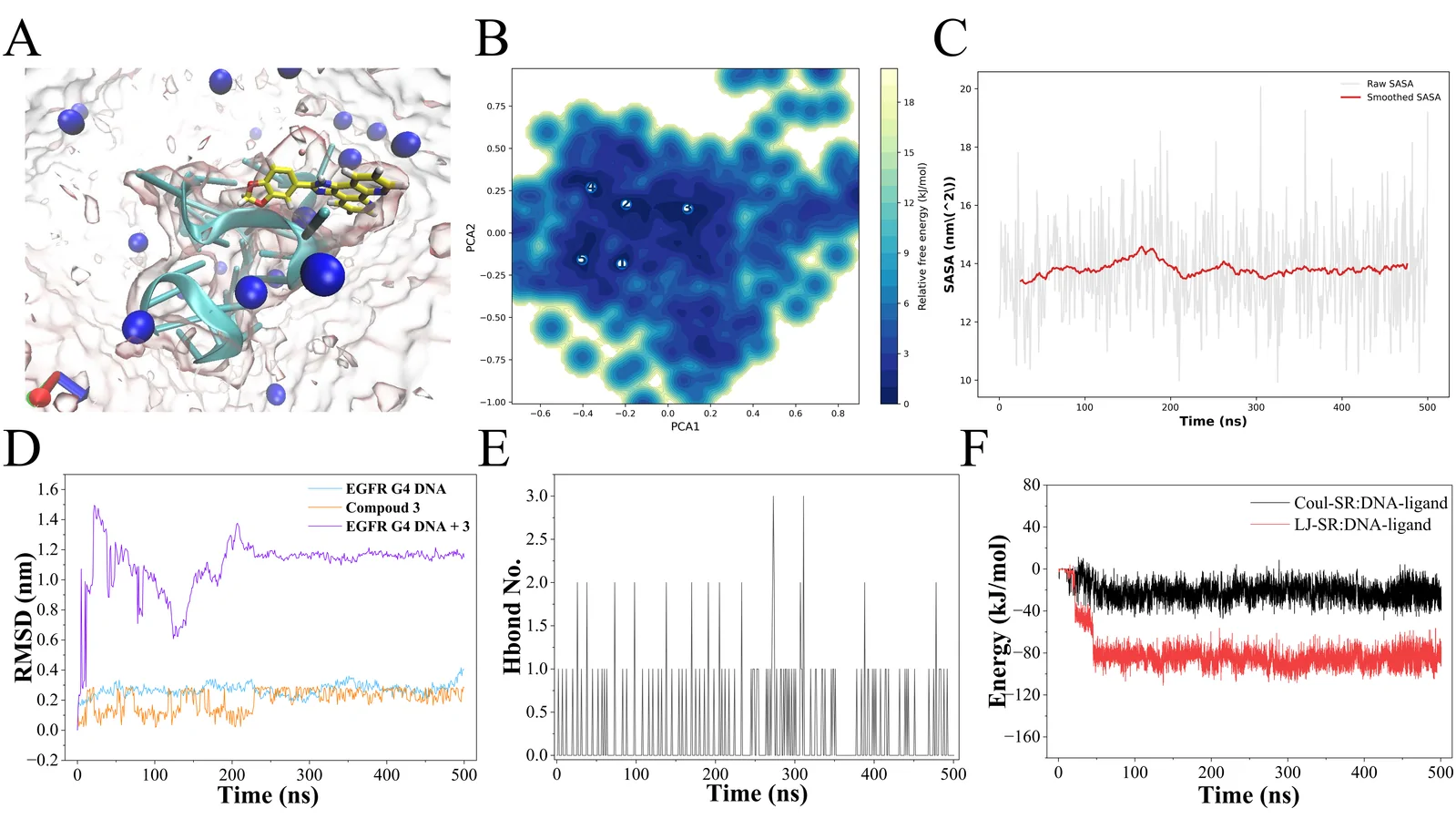
Molecular dynamics simulation analysis. (**A**) Representative MD simulation snapshot of compound **3** bound to the EGFR G-quadruplex DNA. (**B**) Two-dimensional free energy landscape (FEL) of the compound **3**–EGFR G-quadruplex DNA complex, revealing five metastable conformational basins. (**C**) Time-evolution of the solvent-accessible surface area (SASA) of EGFR G-quadruplex DNA throughout the simulation trajectory. (**D**) Root mean square deviation (RMSD) of the free EGFR G-quadruplex DNA, free compound **3**, and the compound **3**–EGFR G-quadruplex DNA complex over the 500 ns simulation. (**E**) Number of hydrogen bonds formed between compound **3** and the EGFR G-quadruplex DNA during the simulation. (**F**) Time-dependent changes in the short-range van der Waals (LJ-SR) and Coulombic (Coul-SR) interaction energies for the compound **3**–EGFR G-quadruplex DNA complex.

To evaluate the dynamic stability of the compound **3**–EGFR G4 complex, a 500 ns all-atom molecular dynamics (MD) simulation was performed (Figure 3A). Two-dimensional free energy landscape (FEL) analysis revealed a shallow energy surface comprising five metastable states, suggesting a conformational selection or induced-fit mechanism where the ligand stabilizes the core G-tetrad via terminal π-π stacking and electrostatic interactions while preserving loop plasticity, thereby dynamically modulating the G4 conformational ensemble rather than rigidly locking it (Figure 3B). Solvent-accessible surface area (SASA) analysis indicated that the SASA remained confined to 5.7–5.8 nm^2^ throughout the simulation, suggesting a compactly folded state devoid of unfolding or structural disorganization (Figure 3C). RMSD trajectories stabilized at ~1.1 nm after initial fluctuations, substantiating an induced-fit backbone rearrangement (Figure 3D). Energy decomposition further quantified the short-range van der Waals energy (LJ-SR) as −95.09 kJ/mol. In contrast, the Coulombic energy (Coul-SR) was only −23.57 kJ/mol, indicating van der Waals interactions, specifically π-π stacking, as the primary thermodynamic driving force, with polar interactions serving a synergistic auxiliary role through intermittent hydrogen bonds (Figure 3E,F). Collectively, these results demonstrate that compound **3** forms a highly stable complex with EGFR G4 via the cooperative interplay of dominant hydrophobic/van der Waals forces and auxiliary polar contacts.

### 3.5. EGFR G4 DNA Binding Properties

**Figure 4:**
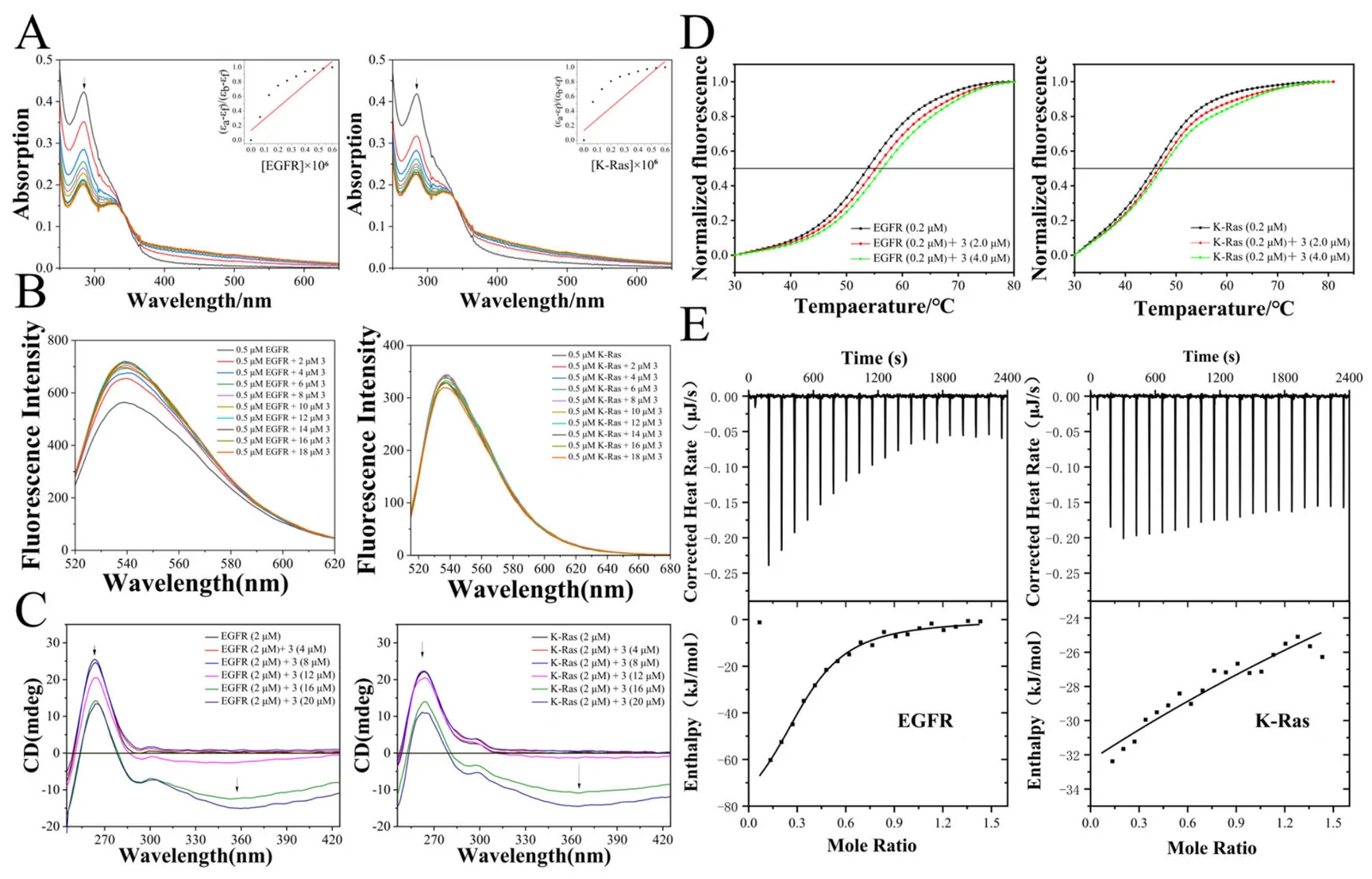
Biophysical characterization of compound **3** binding to EGFR and K-Ras G-quadruplex DNAs. (**A**) Absorption titration of compound **3** (20 μM) with EGFR G4 DNA (0–0.6 μM) (**left**) and K-Ras G4 DNA (0–0.6 μM) (**right**). Insets show the linear fitting plots. (**B**) Fluorescence displacement of thiazole orange (TO, 1 μM) from EGFR G4 DNA (0.5 μM) (**left**) and K-Ras G4 DNA (0.5 μM) (**right**) by compound **3** (0–18 μM). (**C**) Circular dichroism (CD) spectral changes in EGFR G4 DNA (2 μM) (**left**) and K-Ras G4 DNA (2 μM) (**right**) upon the addition of compound **3** at varying concentrations (0–20 μM). (**D**) Fluorescence resonance energy transfer (FRET) profiles depicting the thermal stabilization of EGFR G4 DNA (0.2 μM) (**left**) and K-Ras G4 DNA (0.2 μM) (**right**) by compound **3** at concentrations of 0, 2, and 4 μM. (**E**) Isothermal titration calorimetry (ITC) thermograms (**top**) and the corresponding integrated binding isotherms (**bottom**) for the titration of compound **3** (10 μM) into EGFR G4 DNA (10 μM) (**left**) and K-Ras G4 DNA (10 μM) (**right**) in Tris-HCl buffer (pH 7.4) at 298 K.

To validate the molecular docking predictions, we evaluated the binding of compound **3** to EGFR and K-Ras G4 DNA using UV-Vis absorption and thiazole orange (TO) fluorescence displacement assays. In UV-Vis titration (Figure 4A), as the concentration of G4 DNA increased, the characteristic absorption of compound **3** at 260 nm decreased by 52.72% for EGFR G4 and 46.17% for K-Ras G4, exhibiting a typical hypochromic effect indicative of π–π stacking or end-stacking interactions with stronger electronic coupling for EGFR G4. In the TO displacement assay (Figure 4B), with TO pre-bound to G4 DNA, the addition of compound **3** caused a concentration-dependent increase in TO fluorescence for EGFR G4, reaching a maximum enhancement of approximately 23.6% and approaching saturation. This fluorescence enhancement, rather than quenching, suggests that compound **3** binds to an allosteric site and induces a conformational rearrangement that favors TO intercalation without directly displacing the probe [23]. The fluorescence intensity of TO in the K-Ras G4 system remained essentially unchanged, indicating that compound **3** does not interact effectively with this G4 structure. These data collectively demonstrate that compound **3** displays higher affinity for EGFR G4 and engages a unique allosteric recognition mode.

Circular dichroism (CD) spectroscopy and FRET melting assays further characterized the conformational and thermal stabilization effects. Free EGFR and K-Ras G4 both displayed a characteristic positive Cotton peak around 260 nm, diagnostic of a parallel G4 topology (Figure 4C). With increasing concentration of compound **3**, the intensity of this peak progressively decreased. At the same time, a new broad negative Cotton band emerged in the 350–370 nm region, attributed to exciton coupling from the ligand chromophore terminally stacking within the chiral G4 pocket [24]. FRET melting (Figure 4D) showed that 4.0 μM of compound **3** increased the Tm of EGFR G4 by 2.56 °C, compared with only 1.32 °C for K-Ras G4, a difference of approximately 2-fold. Although Tm shifts were concentration-dependent for both G4s, the stabilization was markedly stronger for EGFR G4, with ΔTm values moderate compared with those of classical G4 ligands such as PDS and Pyridostatin, consistent with the moderate binding affinity of compound **3** [25]. Collectively, the CD and FRET data support that compound **3** selectively stabilizes EGFR G4 through an end-stacking mechanism that induces conformational rearrangement.

Isothermal titration calorimetry (ITC) provided a complete thermodynamic profile (Figure 4E). Compound **3** bound EGFR G4 with a Kd of 102 nM (ΔG = −39.93 kJ/mol) and K-Ras G4 with a Kd of 14.9 μM (ΔG = −27.58 kJ/mol), corresponding to approximately 146-fold selectivity (Table S3). Both interactions were enthalpy-driven (ΔH = −91.6 and −95.43 kJ/mol, respectively) with unfavorable entropy changes (TΔS = −51.73 and −67.85 kJ/mol), indicating that hydrogen bonding, electrostatics, and van der Waals forces dominate recognition, accompanied by reduced conformational flexibility and solvent ordering [26]. The binding stoichiometry n differed fundamentally: K-Ras exhibited *n* ≈ 7.6, suggesting multiple ligands bind non-specifically to a single G4 surface, whereas EGFR showed *n* ≈ 0.33, implying that three G4 molecules associate with one ligand molecule and strongly indicating that compound **3** induces multimerization or end-to-end stacking of EGFR G4 [27]. This unusual binding mode and thermodynamic signature underpin the high selectivity of compound **3**, which achieves high-affinity EGFR G4 recognition through steric and electrostatic complementarity provided by the 6-bromopiperonyl group and the phenanthroline-imidazole scaffold, engaging bromine-mediated halogen bonding, methylenedioxy-based hydrogen bonding, and end-stacking that promotes G4 multimerization.

### 3.6. Structure Activity Relationships

The antitumor activity and normal cytotoxicity evaluation against U87-MG, LN229, A549, and MCF-7 cancer cells, with HMC3 microglia cells (Table 1), revealed that compound **3** exhibited the most favorable biological selectivity profile (IC_50_ = 0.49 ± 0.01 μM against U87-MG, 0.79 ± 0.01 μM against A549; safety index = 14.18), consistent with its superior docking scores and binding selectivity indices for EGFR and K-Ras G4. Compound **1** also showed potent activity against U87-MG and MCF-7 cells (IC_50_ = 0.35 ± 0.005 and 0.72 ± 0.03 μM; safety index = 5.54), with higher lipophilicity (log P = 2.12) facilitating membrane penetration but weaker G4 selectivity. Compounds **2** and **4** displayed extremely low safety indices (0.08 and 0.10), suggesting pronounced non-specific cytotoxicity, while compounds **5** and **6** were weakly active (IC_50_ > 10 μM), likely due to poor membrane permeability (log P = 1.27 and 1.15) [28]. Integrative SAR analysis indicated that the methylenedioxy bridge and bromine atom of the 6-bromopiperonyl group cooperatively provide balanced π-stacking, halogen bonding, and hydrogen bonding while maintaining moderate lipophilicity (log P = 1.60), thereby precisely tuning G4-targeted binding, cell permeability, and selective toxicity, and establishing compound **3** as the most promising anti-glioblastoma candidate for further mechanistic investigation.

In addition, we included comparative data for the reference G4 stabilizer APTO-253 across the U87-MG, LN229, MCF-7, A549, and HMC3 cell lines. The results demonstrate that compound **3** exhibits superior or comparable antiproliferative activity across all tested cell lines, with markedly enhanced tumor cell selectivity. In glioblastoma cells, compound **3** showed an IC_50_ of 0.49 ± 0.01 μM against U87-MG cells, approximately 18-fold more potent than APTO-253 (IC_50_ = 8.97 ± 0.47 μM). In LN229 cells, compound **3** yielded an IC_50_ of 4.85 ± 0.33 μM, whereas APTO-253 was essentially inactive (IC_50_ > 10 μM). Against A549 lung cancer cells, compound **3** was roughly 8.5-fold more potent (IC_50_ = 0.79 μM versus 6.70 μM for APTO-253), and it also outperformed APTO-253 in MCF-7 breast cancer cells (IC_50_ = 5.46 μM versus >10 μM). Importantly, compound **3** displayed a markedly superior safety index (SI = 14.18) relative to APTO-253 (SI = 2.14), indicating a substantially wider therapeutic window. Computationally, all new compounds maintained low duplex DNA affinity, with compound **3** exhibiting the most favorable profile. Its binding energies were −10.16 kcal/mol for EGFR G4 and −9.27 kcal/mol for K-Ras G4, with a dsDNA affinity of only −5.55 kcal/mol. The resulting selectivity indices of 4.61 and 3.72 kcal/mol far exceed those of APTO-253 (0.52 and 2.29 kcal/mol), consistent with the experimental selectivity trends. Together, these experimental and computational comparisons highlight the marked improvements of compound **3** over APTO-253 in potency, tumor selectivity, and G4 discrimination, providing a stronger context for the development of this series.

### 3.7. DNA Damage-Mediated Tumor Cell Death Induced by 3

**Figure 5:**
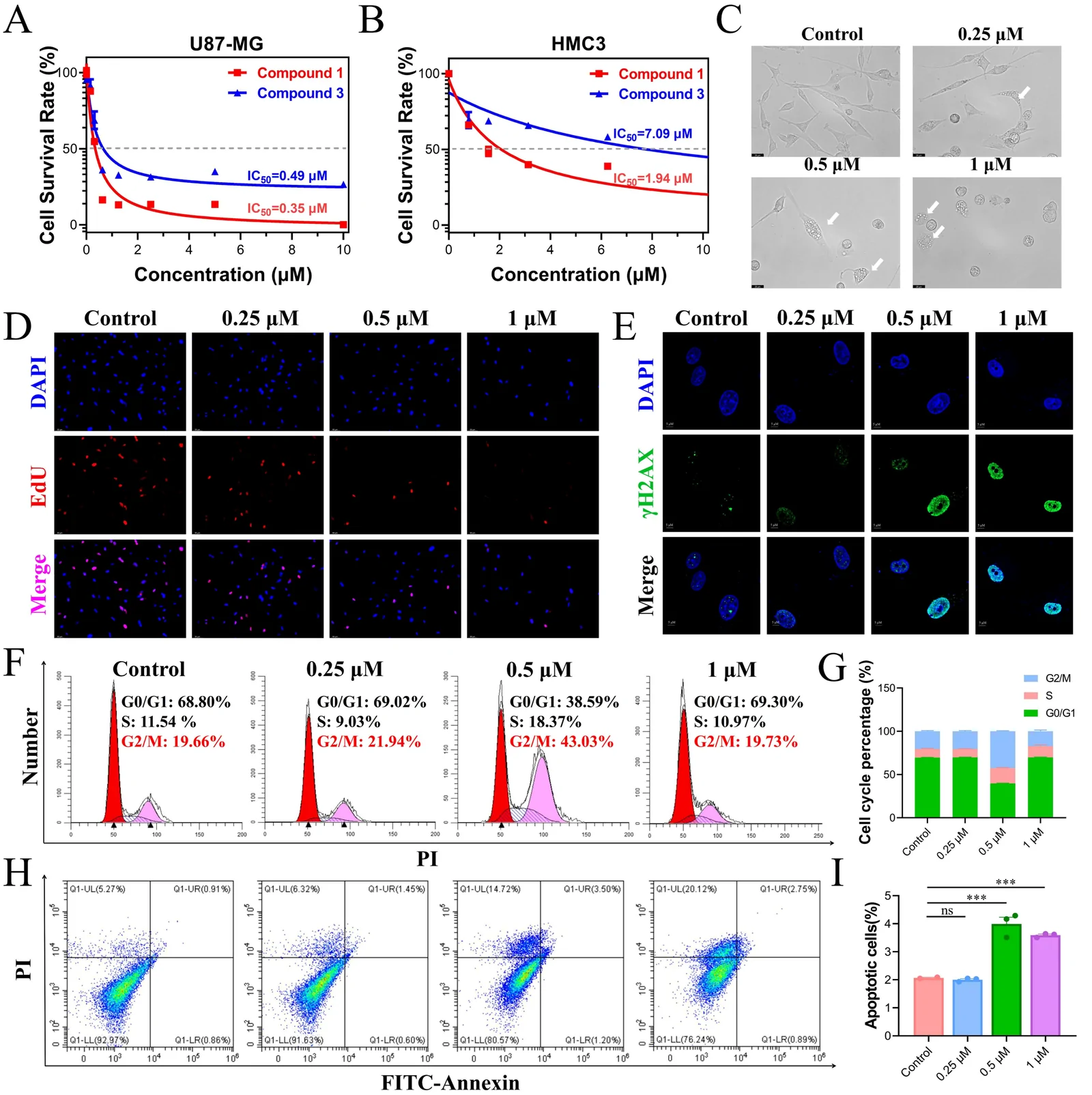
In vitro activity and mechanism of compound **3**. (**A**,**B**) Viability of U87-MG and HMC3 cells treated with compound **1** or **3** for 72 h (IC_50_ values indicated). (**C**–**E**) U87-MG cells treated with compound **3** (0, 0.25, 0.5, 1 μM, 72 h). (**C**) Morphology (white arrows: shrinkage). (**D**) EdU proliferation assay (red) with DAPI (blue). Scale bar, 50 μm. (**E**) γH2AX immunofluorescence (green) for DNA damage. (**F**,**G**) Flow cytometric cell cycle analysis and quantification. (**H**,**I**) Annexin V/PI apoptosis analysis and quantification. Data: mean ± SEM (*n* = 3). *** *p* < 0.001 vs. control; ns, not significant (one-way ANOVA).

Dose–response experiments showed that both compound **1** and compound **3** inhibited U87-MG proliferation in a concentration-dependent manner, with compound **1** exhibiting more potent cytotoxicity (IC_50_ = 0.35 μM; viability < 0.2% at 10 μM) and compound **3** showing relatively moderate inhibition (IC_50_ = 0.49 μM; ~26.53% residual viability at 10 μM) (Figure 5A,B). Toxicity assessment in HMC3 cells revealed a striking selectivity difference: compound **1** caused near-complete HMC3 cell death (4.64% viable at 25 μM), whereas compound **3** retained 33.28% viability, confirming a wider therapeutic window. Morphological observation (Figure 5C) showed that compound **3** progressively induced autophagic vacuole formation from 0.25 μM, with extensive cytoplasmic vacuolization and loss of adherence at 1 μM, indicative of escalating autophagy leading to irreversible cell damage [29].

To define the underlying molecular mechanism, we evaluated DNA integrity, cell cycle progression, and apoptosis. The EdU incorporation assay (Figure 5D and Figure S20) showed a concentration-dependent decline in DNA synthesis, with the EdU-positive rate decreasing from 82.43% at 0.25 μM to 16.44% at 1 μM, indicating near-complete proliferative arrest. γH2AX immunofluorescence (Figure 5E) revealed that compound **3** induced progressively accumulating DNA double-strand breaks (DSBs), with intense pan-nuclear γH2AX foci at 1 μM [30]. Cell cycle analysis (Figure 5F,G) showed that 0.5 μM compound **3** triggered pronounced G2/M arrest (G2/M population 43.03% versus 19.66% in controls), whereas at 1 μM, the cycle distribution appeared to recover, likely reflecting the selective detachment of lethally damaged cells, leaving only a residual adherent population [31,32]. Apoptosis detection (Figure 5H,I) indicated that total apoptosis increased to 4.70% at 0.5 μM, predominantly late apoptosis, but declined to 3.64% at 1 μM, suggesting that high concentrations drive a fraction of cells toward necrosis or other non-apoptotic death pathways [33]. Taken together, compound **3** induces severe DNA damage, triggers G2/M arrest, and promotes a mixed mode of apoptosis and necrosis in a concentration-dependent manner.

### 3.8. Mitochondria-Derived Oxidative Stress Induced by 3

**Figure 6:**
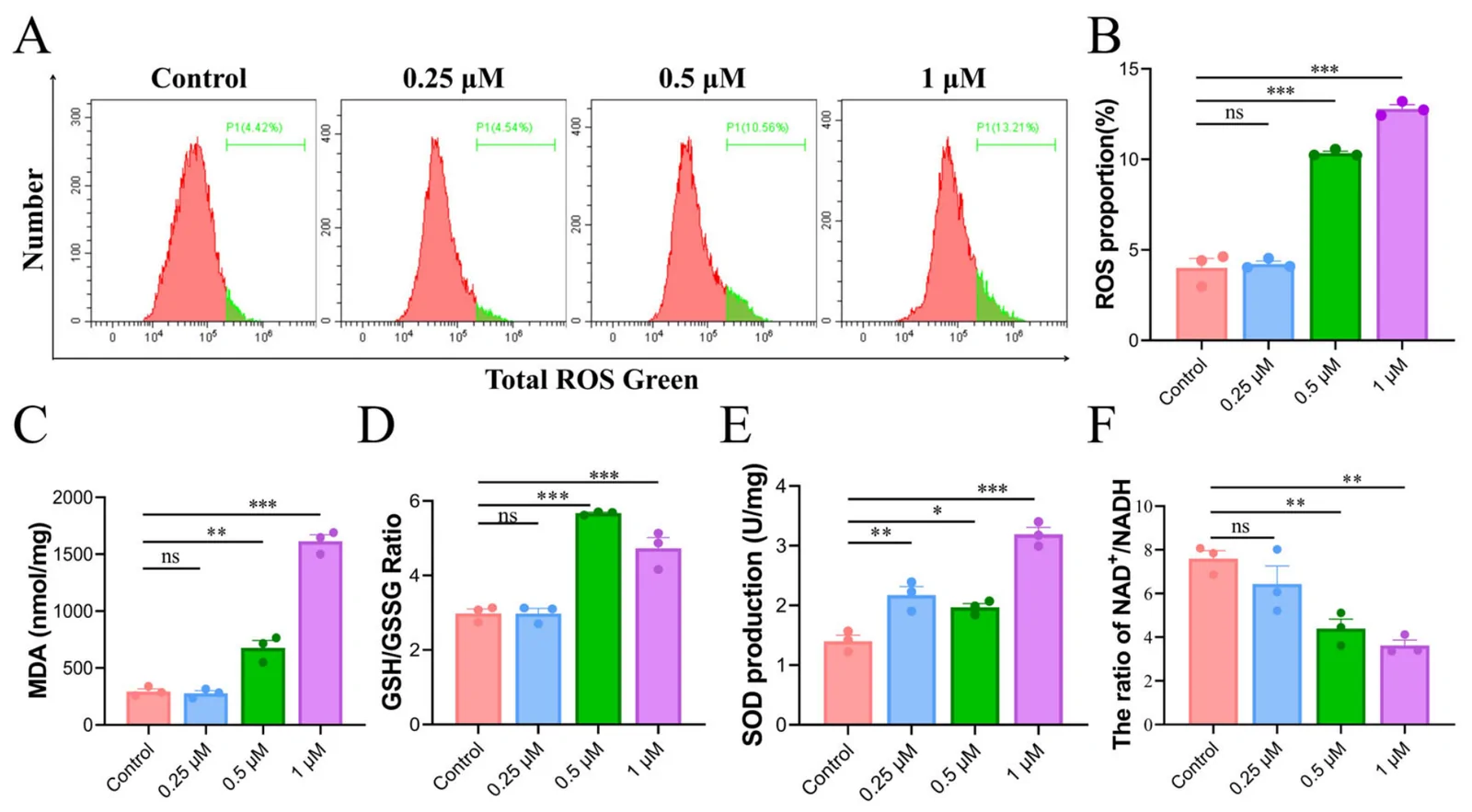
Compound **3** induces oxidative stress and disrupts redox homeostasis in U87-MG cells. (**A**) Flow cytometric analysis of intracellular total ROS levels using Total ROS Green probe in U87-MG cells treated with compound **3** at the indicated concentrations (0, 0.25, 0.5, and 1 μM). (**B**) Quantitative statistical analysis of the ROS-positive proportion (%) derived from (**A**). (**C**–**F**) Evaluation of oxidative stress-related biochemical parameters in U87-MG cells following treatment with compound **3**, including malondialdehyde (MDA) content (**C**), GSH/GSSG ratio (**D**), superoxide dismutase (SOD) production (**E**), and NAD^+^/NADH ratio (**F**). Data are presented as mean ± SEM (*n* = 3). * *p* < 0.05, ** *p* < 0.01, *** *p* < 0.001 vs. the control group; ns indicates no significant difference (one-way ANOVA).

Given the frequent association between DNA damage and redox imbalance, we examined whether oxidative stress contributes to the cytotoxicity of compound **3** [34]. Total ROS, measured with the DCFH-DA probe (Figure 6A,B), increased modestly from 4.42% in controls to 13.21% at 1 μM (*p* < 0.001). In contrast, the MitoSOX assay revealed a pronounced burst of mitochondrial ROS (mtROS), with the positive rate surging from 3.71% to 43.58% at 1 μM, indicating that mitochondria are the primary source of compound **3**-induced oxidative stress. This mtROS overproduction was accompanied by extensive lipid peroxidation, as reflected by a 5.49-fold increase in MDA content at 1 μM (1613.59 nmol/mg versus 293.64 nmol/mg in controls; *p* < 0.001) (Figure 6C), indicating severe oxidative damage to membranes.

Analysis of antioxidant defenses revealed a biphasic response. The GSH/GSSG ratio (Figure 6D) increased from 2.98 in controls to 5.67 at 0.5 μM (*p* < 0.001), suggesting a compensatory upregulation of glutathione reserves, but declined to 4.73 at 1 μM, consistent with GSH depletion exceeding regenerative capacity. SOD activity (Figure 6E) rose from 1.40 U/mg to 3.19 U/mg at 1 μM (*p* < 0.001), while the NAD^+^/NADH ratio (Figure 6F) decreased from 7.59 to 3.62 (*p* < 0.01), reflecting severe redox potential collapse and energetic crisis. Collectively, these data indicate that compound **3** triggers a mitochondria-driven ROS burst and lipid peroxidation concurrent with multi-organelle damage; at moderate concentrations, compensatory antioxidant responses are mounted, but at high concentrations, enzymatic and non-enzymatic defenses become decompensated, culminating in redox collapse and irreversible cell death [35,36].

### 3.9. Mitochondria-Lysosome-Endoplasmic Reticulum Concurrent Multi-Organelle Damage Induced by 3

**Figure 7:**
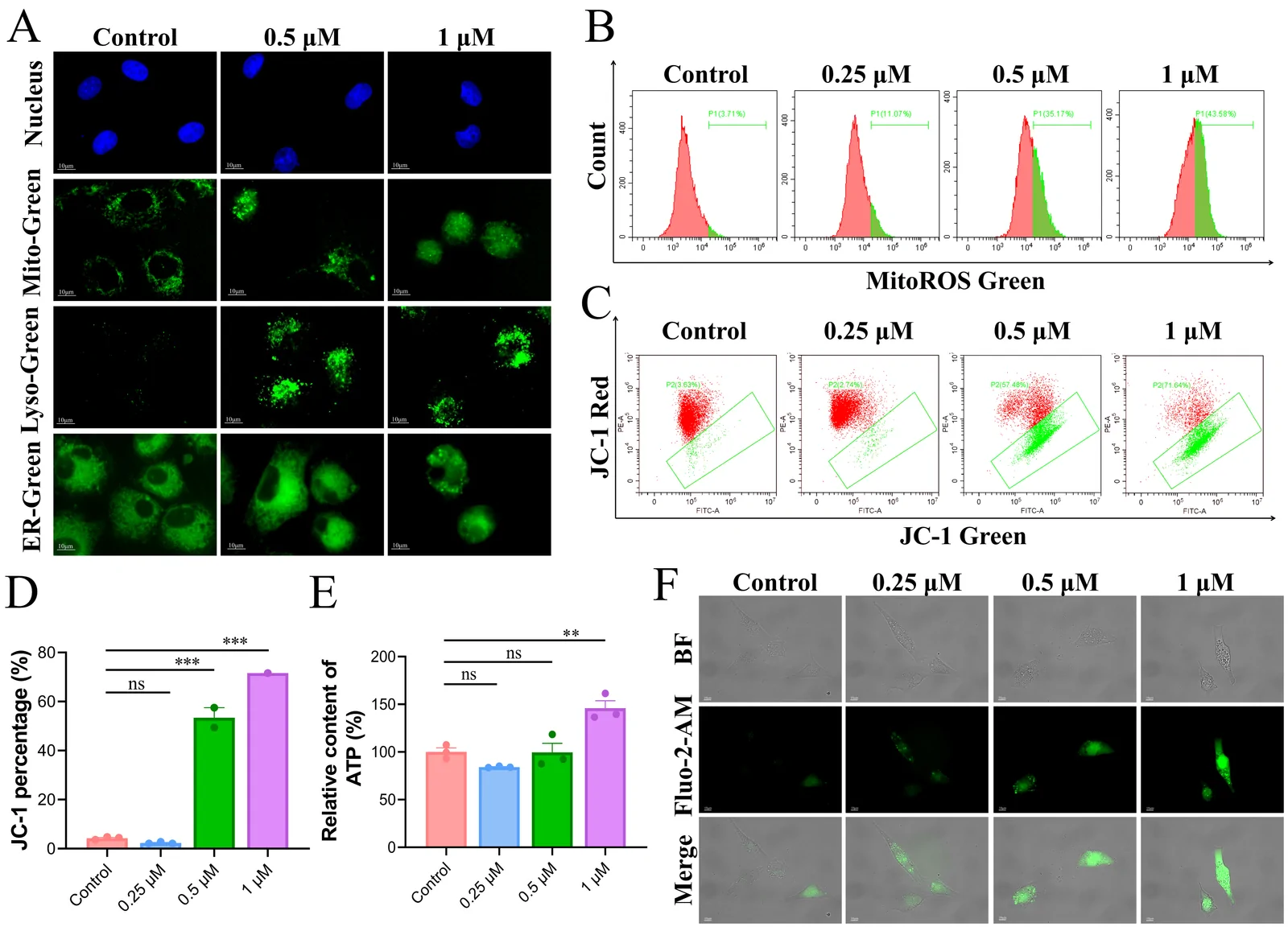
Compound **3** induces mitochondrial dysfunction and perturbation of calcium homeostasis in U87-MG cells. (**A**) Fluorescence images of mitochondria (Mito-Green), lysosomes (Lyso-Green), and ER (ER-Green) after compound **3** treatment. Nuclei were stained with DAPI (blue). (**B**) Flow cytometry of mitochondrial ROS (MitoROS). (**C**,**D**) Mitochondrial membrane potential (ΔΨm) assessed by JC-1. (**C**) Representative scatter plots. (**D**) Quantification of JC-1-positive cells. (**E**) Relative ATP content. (**F**) Intracellular Ca^2+^ detection with Fluo-2 AM (brightfield, fluorescence, merge). Data: mean ± SEM. ** *p* < 0.01, *** *p* < 0.001 vs. control; ns, not significant (one-way ANOVA).

To systematically characterize the organellar damage induced by compound **3**, we employed immunofluorescence staining of nuclei, mitochondria, lysosomes, and the ER (Figure 7A). Control cells displayed intact organelle morphology. At 0.5 μM, mitochondria underwent fragmentation into dispersed puncta with local aggregation, lysosomal signals intensified and expanded, and the ER showed mild disorganization. At 1 μM, nuclei became pyknotic with crescent-shaped deformity; the mitochondrial network collapsed into granular aggregates; lysosomes merged into sheet-like perinuclear distributions; and the ER reticular structure was replaced by pronounced vacuolization, demonstrating concentration-dependent, concurrent multi-organelle disintegration [37].

Moreover, the MitoSOX detection (Figure 7B) confirmed that mtROS increased from 3.71% to 43.58% at 1 μM, indicating progressive ETC dysfunction [38]. JC-1 staining (Figure 7C,D) showed that the high-MMP population declined from 96.37% to 42.52% at 0.5 μM and to 28.36% at 1 μM, characteristic of MMP collapse, with a significant negative correlation between MMP loss and mtROS elevation. ATP levels paradoxically rose to 145.90% of control at 1 μM (Figure 7E), suggesting a stress-induced glycolytic compensation rather than mitochondrial recovery [35]. Fluo-2-AM imaging (Figure 7F) revealed concentration-dependent cytosolic Ca^2+^ overload, closely linked to mitochondrial dysfunction, lysosomal membrane permeabilization, and ER disintegration, forming a self-amplifying pathological network that drives glioblastoma cell death [39,40].

**Figure 8:**
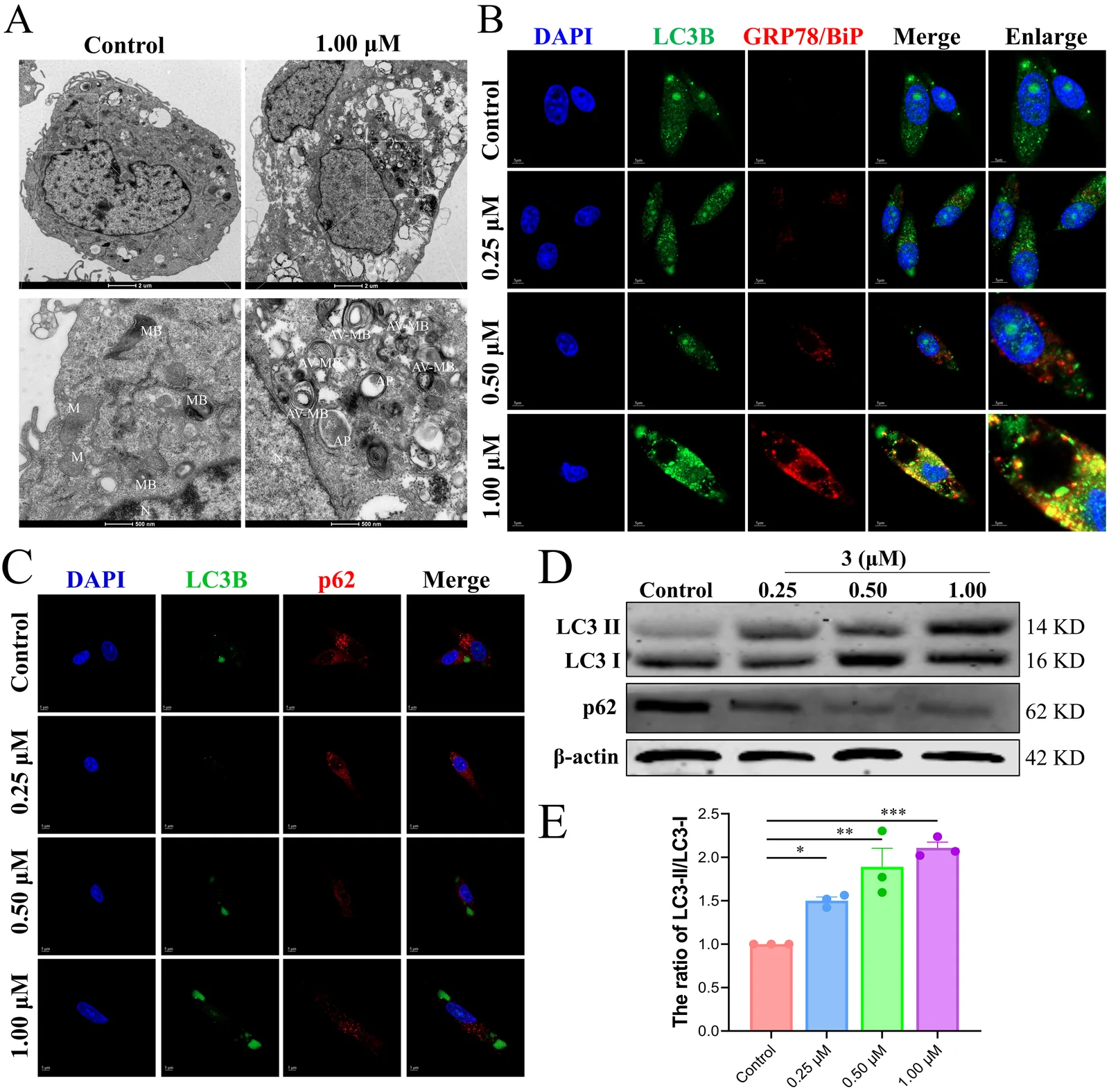
Compound **3** induces autophagy and endoplasmic reticulum (ER) stress in U87-MG cells. (**A**) TEM images showing autophagosomes and autolysosomes in U87-MG cells (control and 1 μM). (**B**) Immunofluorescence co-staining of LC3B (green) and GRP78/BiP (red) with DAPI (blue) for nuclear counterstaining in cells treated with compound **3** at the indicated concentrations. (**C**) Immunofluorescence of LC3B (green) and p62 (red) after compound **3** treatment. (**D**) Western blot of LC3B (LC3-I/II) and p62 in U87-MG cells, with β-actin as loading control. (**E**) Quantification of LC3-II/LC3-I ratio from (**D**). Data: mean ± SEM (*n* = 3). * *p* < 0.05, ** *p* < 0.01, *** *p* < 0.001 vs. control (one-way ANOVA).

Furthermore, Bio-TEM (Figure 8A) provided ultrastructural validation, showing pyknotic nuclei with membrane invagination, swollen mitochondria with blurred cristae and matrix vacuolization, and abundant autophagosomes and autophagic vacuoles containing multilamellar myelin bodies, indicative of overwhelmed lysosomal degradation of damaged membranous organelles. GRP78/BiP–LC3B co-localization (Figure 8B) demonstrated that ER stress and autophagy were simultaneously activated at 0.5 μM and became extensively coupled at 1 μM, with dense perinuclear overlap [41]. LC3B–p62 co-localization (Figure 8C) confirmed that p62 was efficiently cleared at higher concentrations, indicating that compound **3** promotes complete autophagic flux rather than blocking degradation. Western blot analysis further corroborated these findings (Figure 8D), as the concentration of compound **3** increased, a marked accumulation of LC3B-II was observed, whereas LC3B-I levels remained largely unchanged, reflecting enhanced autophagosome formation without apparent alteration in LC3B-I expression. Concurrently, p62 protein levels declined in a concentration-dependent manner, confirming sustained degradation of autophagic substrates [42]. Consistently, the LC3B-II/LC3B-I ratio exhibited a significant concentration-dependent increase (Figure 8E), quantitatively reinforcing the notion that compound **3** potently induces autophagic flux. Collectively, these results demonstrate that compound **3** triggers ER stress-coupled autophagy and drives complete autophagic degradation in a concentration-dependent fashion. Although robust autophagy was activated in response to stress, it ultimately failed to restore homeostasis and instead cooperated with apoptotic and necrotic pathways to execute cell death.

### 3.10. Suppression of Migration and Invasion of U87-MG Cells by 3

**Figure 9:**
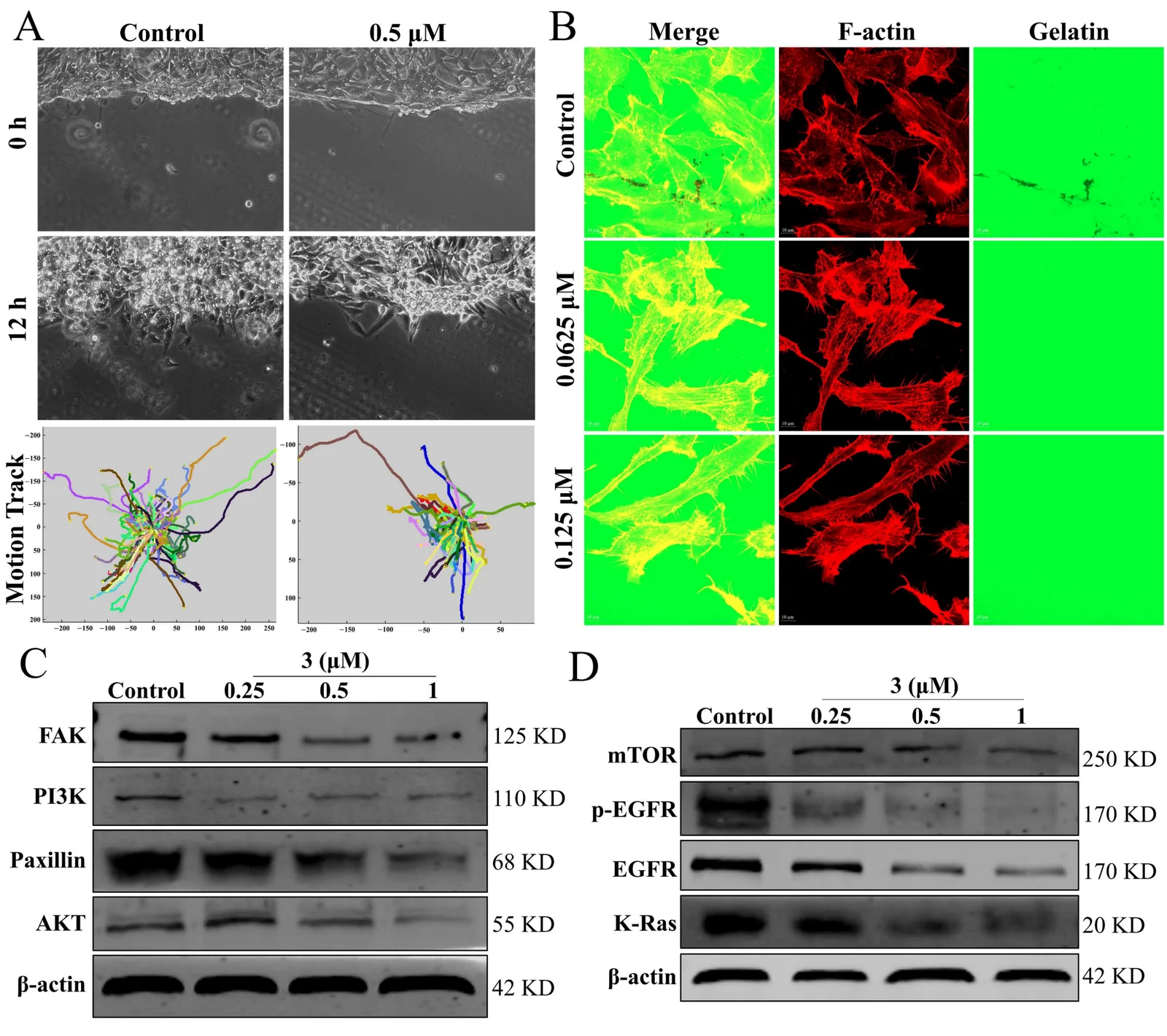
Compound **3** suppresses migration, invasion, and related signaling in U87-MG cells. (**A**) Wound healing (**top**) and migration trajectories (**bottom**) after 0 or 0.5 μM compound **3** for 12 h. (**B**) FITC-gelatin invasion assay (green: undegraded gelatin, red: F-actin) with 0, 0.0625, 0.125 μM compound **3**. (**C**) Western blot of FAK, PI3K, Paxillin, AKT, (**D**) and mTOR, p-EGFR, EGFR, K-Ras, after compound **3** treatment (0–1 μM); β-actin as loading control. Data: mean ± SEM.

Given that the high malignancy of GBM critically depends on tumor cell migration and invasion, we evaluated the effects of compound **3** on these properties. In the wound-healing assay (Figure 9A), 0.5 μM compound **3** markedly reduced cell migration into the scratch area compared with the vehicle control. The FITC-gelatin degradation assay with F-actin staining (Figure 9B) further showed that compound **3**, even at 0.0625 μM, substantially decreased cell spreading and completely abolished gelatin degradation, indicating potent suppression of ECM proteolytic activity. At 0.125 μM, the F-actin stress fiber network was disrupted and disorganized, with loss of pseudopodial structures, confirming that compound **3** impairs cytoskeletal integrity and matrix-degrading capacity at sub-cytotoxic concentrations [14].

Western blot analysis (Figure 9C) demonstrated that compound **3** downregulated FAK, PI3K, Paxillin, and AKT in a concentration-dependent manner, with AKT nearly undetectable at 1 μM, consistent with the observed stress fiber disruption and pseudopodial loss. Furthermore, based on the high affinity of compound **3** for EGFR and K-Ras promoter G4 structures, we validated target engagement at the protein level (Figure 9D). Compound **3** treatment led to a concentration-dependent decrease in p-EGFR, total EGFR, K-Ras, and mTOR, with p-EGFR and K-Ras virtually disappearing at 1 μM. These results indicate that compound **3** suppresses EGFR phosphorylation and downregulates K-Ras–mTOR signaling, thereby blocking proliferation, survival, migration, and invasion pathways at the level of signal transduction, which mechanistically aligns with its antitumor efficacy observed in cellular assays [43].

### 3.11. In Vivo Antitumor Activity Assessment

**Figure 10:**
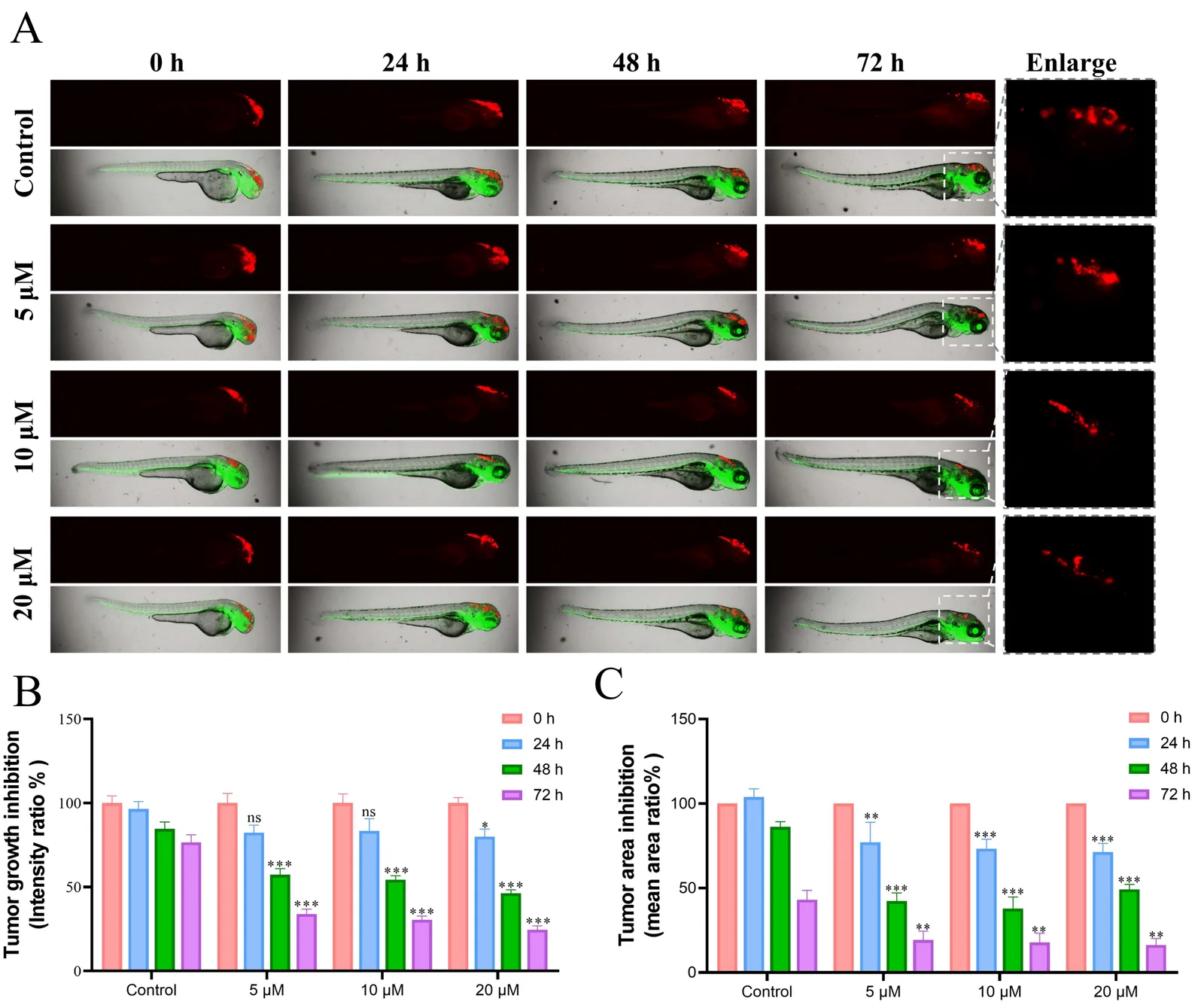
Inhibition of compound **3** on the proliferation and metastasis of U87-MG cells in a zebrafish glioblastoma in situ model. (**A**) Representative fluorescence microscopy images showing the growth and metastasis of U87-MG cells (red fluorescence) in the zebrafish glioblastoma in situ model in the absence or presence of compound **3** at indicated concentrations (0, 5, 10, and 20 μM) for 0, 24, 48, and 72 h, with enlarged views of the tumor regions. (**B**) Quantitative analysis of tumor growth inhibition (intensity ratio %) of U87-MG cell xenografts in the zebrafish head (*n* = 6/group). (**C**) Quantitative analysis of tumor area inhibition (mean area ratio %) of U87-MG cell xenografts in the zebrafish head (*n* = 6/group). Data are presented as means ± SEM. Statistical significance was determined by one-way analysis of variance (ANOVA). Values were significantly different from the control group at * *p* < 0.05, ** *p* < 0.01, and *** *p* < 0.001; ns: no significance.

To evaluate the in vivo anti-glioblastoma activity of Compound **3**, we established an orthotopic U87-MG glioma xenograft model in the zebrafish brain [14]. DiI-labeled U87-MG cells were injected into the brain of zebrafish larvae, and the tumor-bearing larvae were exposed to embryo medium containing different concentrations of Compound **3** (5, 10, and 20 µM) for 72 h. Tumor growth was quantified by measuring changes in tumor fluorescence intensity and area. At 0 h, clear, uniformly distributed DiI fluorescence signals of comparable intensity were observed in the brain region of all groups (Figure 10A), indicating successful tumor cell engraftment and comparable baseline levels among groups. After 72 h, the tumor fluorescence intensity in the control group decreased by 23.35% (Figure 10B), and the fluorescent area decreased by 56.89% (Figure 10C) compared with 0 h, suggesting a certain degree of spontaneous reduction in tumor burden under the pressure of innate immune rejection and the xenogeneic microenvironment in zebrafish. Compared with the control group, the attenuation of tumor fluorescence signals in the Compound **3**-treated groups was significantly accelerated and concentration-dependent. Following 72 h of treatment with 5 µM Compound **3**, the tumor fluorescence intensity decreased by 66.02% (Figure 10B) and the fluorescent area decreased by 80.77% (Figure 10C) relative to 0 h, with both reductions significantly greater than those in the control group (*p* < 0.01), preliminarily demonstrating its in vivo antitumor efficacy. When the concentration was increased to 10 µM, the fluorescence intensity decreased by 69.56% (Figure 10B) and the fluorescent area by 82.21% (Figure 10C), further enhancing tumor inhibition (*p* < 0.01 vs. control). At 20 µM Compound **3**, the fluorescence signal became very weak and was scattered, with fluorescence intensity reduced by 75.41% (Figure 10B) and fluorescent area reduced by 83.75% (Figure 10C) compared with 0 h; the reduction in intensity was 3.23-fold, and the reduction in area was 1.47-fold that of the control group. In conclusion, Compound **3** strongly inhibited U87-MG tumor growth in a concentration-dependent manner in the zebrafish orthotopic brain glioma model, demonstrating significant in vivo anti-glioblastoma activity.

## 4. Discussion

Given the limited efficacy of EGFR kinase inhibitors in glioblastoma, owing largely to poor brain penetration and acquired resistance, targeting the EGFR promoter G-quadruplex (G4) offers a transcription-level intervention that bypasses these limitations. In this study, we rationally designed a focused series of phenanthroimidazole derivatives by systematic bioisosteric modification of the clinical-stage G4 ligand APTO-253 [6]. Among them, compound **3**, bearing a 6-bromopiperonyl substituent, emerged as a highly selective EGFR G4 stabilizer with nanomolar affinity and ~146-fold selectivity over K-Ras G4. Direct comparison with APTO-253 demonstrated that compound **3** is approximately 18-fold more potent against U87-MG cells and retains substantial activity in LN229 cells, whereas APTO-253 is essentially inactive in LN229 cells. It also exhibits a markedly wider safety margin in normal HMC3 microglia. These experimental gains are supported by computational selectivity indices, indicating that the synergistic combination of methylenedioxy hydrogen-bond acceptors and bromine halogen-bond donors effectively redirects the selectivity profile toward EGFR G4 while maintaining favorable drug-like properties.

**Scheme 2:**
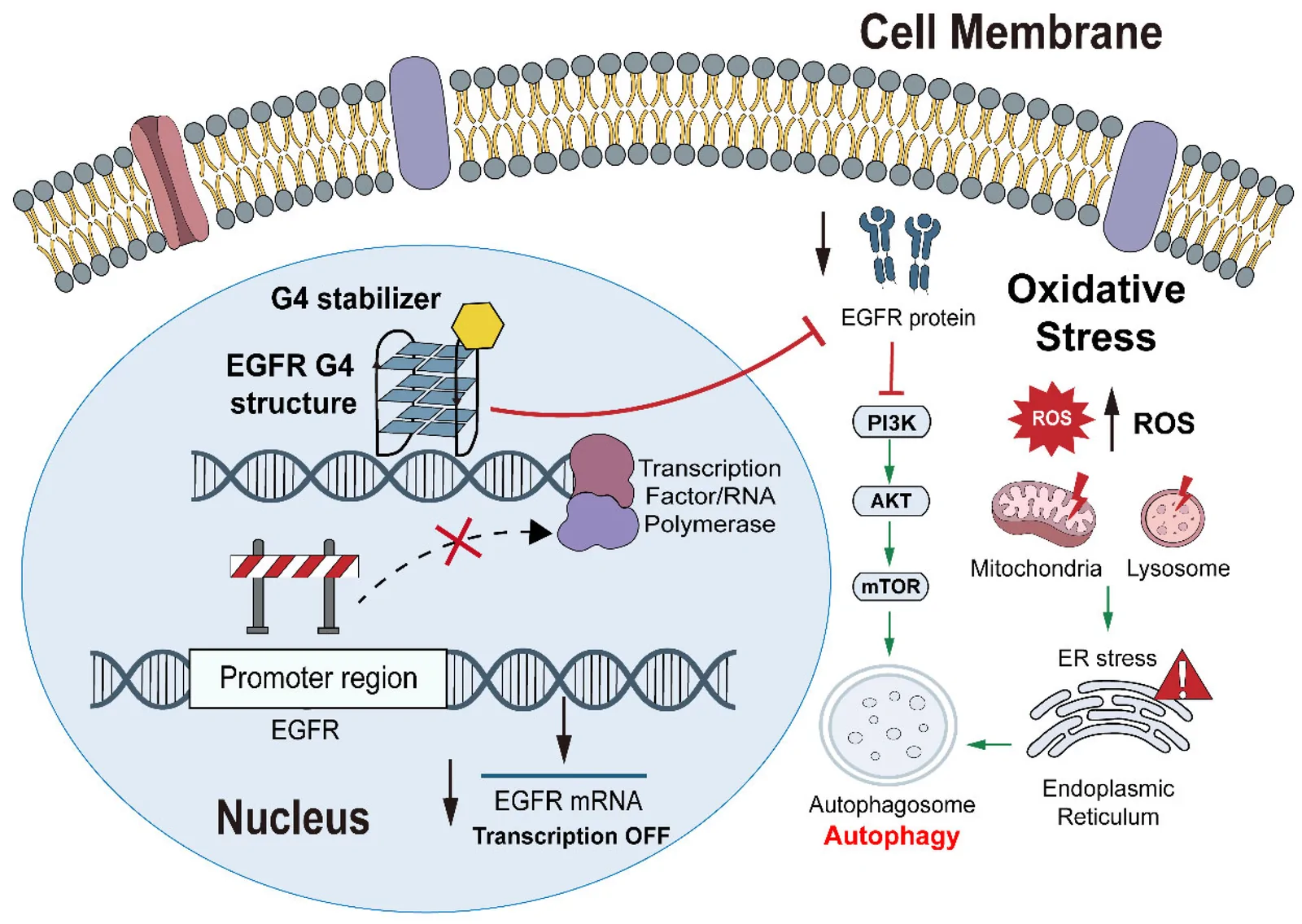
Schematic illustration of G-quadruplex stabilization by specific ligands, resulting in enhanced replication stress and DNA damage that drive transcriptional repression and cancer cell autophagy.

Mechanistically, we provide direct cellular evidence that compound **3** stabilizes intracellular G4 structures and suppresses EGFR transcription in a concentration-dependent manner, as demonstrated by BG4 immunofluorescence and qPCR, respectively. The observed multi-organelle damage, including mitochondrial oxidative stress, lysosomal impairment, and ER stress, occurs concurrently at the examined time points and is associated with the inhibition of migration and invasion programs. While these phenotypic consequences correlate well with EGFR G4 engagement, we emphasize that the current data establish association rather than a strict temporal cascade; dedicated time-course and genetic rescue experiments will be required to resolve the causal hierarchy among these events (Scheme 2). Computational docking and dynamics simulations provided structural hypotheses for the observed binding preferences, but biophysical and cell-based assays ultimately assess biological selectivity and cellular target engagement. The consistent antiproliferative activity across two genetically distinct GBM cell lines further supports the broader applicability of the proposed mechanism beyond a single cellular context.

Despite the promising findings, several limitations should be acknowledged. Direct target engagement of compound **3** with the endogenous EGFR promoter G4, and the causal link between G4 stabilization and the observed organelle damage, await further confirmation using techniques such as ChIP/CETSA and genetic rescue experiments. The G4 selectivity assessment was performed on a limited panel of sequences, and the mechanistic model was primarily established in U87-MG cells, highlighting the need for genome-wide selectivity profiling and validation in broader GBM contexts [44]. In vivo efficacy was examined only in a zebrafish xenograft model, and mammalian orthotopic studies with comprehensive pharmacokinetic and brain penetration evaluations will be essential for translational advancement. Acknowledging these limitations does not diminish the current findings but provides a candid framework for future investigations.

## 5. Conclusions

This study performed systematic bioisosteric modifications at the C2 aryl position of the phenanthroline-imidazole scaffold and identified compound **3** as a nanomolar-affinity (Kd = 102 nM), highly selective (approximately 146-fold) EGFR G4 stabilizer guided by computer-aided drug design. This ligand binds to and induces EGFR G4 multimerization via a distinctive end-stacking mode, exhibiting potent antiproliferative activity against U87-MG glioblastoma cells (IC_50_ = 0.49 μM) and a favorable safety margin (safety index of 14.18). Mechanistically, compound **3** induces DNA damage and mitochondrial oxidative stress, triggering concurrent multi-organelle damage involving mitochondria, lysosomes, and the endoplasmic reticulum, while concurrently suppressing tumor cell migration and invasion. This study provides a new lead molecule for EGFR G4-targeted glioblastoma therapy and advances mechanistic understanding of how G4 ligands exert antitumor activity through synergistic multi-organelle damage.

## Figures and Tables

**Table 1:** Inhibitory effects of phenanthroimidazole derivatives against various human cancer and normal cell Lines with their lipophilicity.

Comp.		IC_50_(μM)					
	U87-MG	LN229	MCF-7	A549	HMC3	Safe Index	Log P
**1**	0.35 ± 0.005	>10	0.72 ± 0.03	9.34 ± 0.13	1.94 ± 0.03	5.54	2.12
**2**	8.12 ± 0.097	>10	0.72 ± 0.02	>10	0.62 ± 0.03	0.08	2.13
**3**	0.49 ± 0.01	4.85 ± 0.33	5.46 ± 0.05	0.79 ± 0.01	7.09 ± 0.37	14.18	1.60
**4**	>10	>10	>10	>10	1.26 ± 0.03	0.10	1.85
**5**	>10	>10	>10	>10	11.97 ± 0.82	<1.20	1.27
**6**	8.30 ± 0.11	>10	>10	>10	32.95 ± 1.19	3.97	1.15
APTO-253	8.97± 0.47	>10	>10	6.70 ± 0.44	19.20 ± 1.31	2.14	1.31

Safe Index = IC_50_ (HMC3)/IC_50_ (U87-MG).

## Data Availability

The data presented in this study are available on request from the corresponding authors.

## Supplementary Materials

The following supporting information can be downloaded at: https://www.mdpi.com/article/10.3390/antiox15080984/s1. Table S1: The antibodies used in this study; Table S2: Crystallographic and Unit Cell Parameters for 3; Figure S1: Molecular packing of compound **5** viewed along the a, b, and c crystallographic axes; Figure S2–S7: ESI-MS spectrum of 1–6; Figure S8–S13: The ^1^H NMR spectrum of 1–6; Figure S14–S19: The ^13^C NMR spectrum of 1–6.; Table S3: ITC binding isotherm of 3 (10 μM) titrated into EGFR (10 μM) or K-Ras (10 μM) in Tris-HCl (pH 7.4, 298 K); Table S4–S8: Acid dissociation constant (pKa) data for 1–4 and 6 in 20% (*v*/*v*) ethanol-water solution; Figure S20: Quantitative analysis of EdU positive cell ratio normalized to control; Figure S21: LC3B of original Western blot for three repeats; Figure S22: p62 of original western blot for three repeats; Figure S23: FAK of original Western blot for three repeats; Figure S24: PI3K of original Western blot for three repeats; Figure S25: Paxillin of original western blot for three repeats; Figure S26: AKT of original Western blot for three repeats; Figure S27: mTOR of original western blot for three repeats; Figure S28: p-EGFR of original Western blot for three repeats; Figure S29: EGFR of original western blot for three repeats; Figure S30: K-Ras of original Western blot for three repeats; Figure S31: Immunofluorescence staining of BG4 (red) with DAPI (blue) to evaluate G-quadruplex (G4) stabilization in U87-MG cells treated with compound **3**; Figure S32: Relative EGFR mRNA expression levels in U87-MG cells treated with compound **3** (0, 0.25, 0.5, 1 μM) for 72 h, measured by quantitative real-time PCR. Data are presented as mean ± SEM (*n* = 3). * *p* < 0.05, ** *p* < 0.01, *** *p* < 0.001 vs. control group.

## Abbreviations

The following abbreviations are used in this manuscript:

GBM	Glioblastoma multiforme
G4	G-quadruplex
ITC	Isothermal titration calorimetry
CD	Circular dichroism
EGFR	Epidermal Growth Factor Receptor
K-Ras	Kirsten Rat Sarcoma Viral Oncogene Homolog
c-Myc	Cellular Myelocytomatosis Viral Oncogene Homolog
VEGF	Vascular Endothelial Growth Factor
Bcl-2	B-cell Lymphoma 2
Tel26	Telomeric Repeat Sequence (TTAGGG) × 26
c-Kit1	KIT Proto-oncogene 1
c-Kit2	KIT Proto-oncogene 2
dsDNA	Double-stranded DNA
Bio-TEM	Biological Transmission Electron Microscopy
PDS	Pyridostatin
ROS	Reactive Oxygen Species
GSH	Glutathione
GSSG	Glutathione Disulfide
SOD	Superoxide Dismutase
LAMP1	Lysosomal-Associated Membrane Protein 1
PTU	Phenylthiourea
