# Supplementary material for: Computer-Aided Discovery of EGFR G-Quadruplex Targeting Phenanthroimidazole Derivatives Inducing Concurrent Multi-Organelle Damage in Glioblastoma
Source: Antioxidants (Basel). 2026 Aug 8;15(8):984. doi: 10.3390/antiox15080984 (PMC13509831; doi:10.3390/antiox15080984)
Supplement: Supplementary file 1 [file antioxidants-15-00984-s001.zip › antioxidants-4423783-supplementary.pdf]

Supporting Information
for
Computer-Aided Discovery of EGFR G-Quadruplex Targeting Phenanthroimidazole Derivatives Inducing Concurrent Multi-Organelle Damage in Glioblastoma

1. Materials

Table S1. The antibodies used in this study.

Antibody name	Host species	Cat.No.	Brand
Anti-gamma H2A.X (phospho S139) antibody [9F3]	Mouse	ab26350	Abcam
CD107a / LAMP1 Monoclonal antibody	Mouse	67300-1-Ig	Proteintech
LC3B-Specific Polyclonal antibody	Mouse	18725-1-AP	Proteintech
GRP78/BIP Monoclonal antibody	Mouse	66574-1-Ig	Proteintech
P62/SQSTM1 Monoclonal antibody	Mouse	66184-1-Ig	Proteintech
FAK Polyclonal antibody	Rabbit	12636-1-AP	Proteintech
Anti-PI3 Kinase p110 betaantibody[EPR5515(2)]	Mouse	ab151549	Abcam
Paxillin Polyclonal antibody	Rabbit	22172-1-AP	Proteintech
Anti-pan-AKT(ab8805)	Rabbit	ab8805	Abcam
Anti-EGFR (phospho Y1068) antibody [EP774Y]	Rabbit	ab40815	Abcam
Anti-EGFR antibody [EP38Y]	Rabbit	ab52894	Abcam
mTOR Polyclonal antibody	Mouse	20657-1-AP	Proteintech
KRAS Polyclonal antibody	Rabbit	12063-1-AP	Proteintech
Anti-Mouse IgG H&L (Alexa Fluor® 647)	Mouse	ab150115	Abcam
Anti-Rabbit IgG H&L (Alexa Fluor® 488)	Rabbit	ab150077	Abcam

2. Results

Table S2. Crystallographic and Unit Cell Parameters for 3.

Identification code	ww
Empirical formula	$C_{20}H_{11}BrN_4O_2$
Formula weight	419.23
Temperature/K	150(2)
Crystal system	monoclinic
Space group	$P2_1/c$
a/Å	11.4967(8)
b/Å	22.5415(10)
c/Å	15.7159(8)
α/°	90
β/°	126.575(3)
γ/°	90
Volume/Å^3	3270.8(3)
Z	8
ρ_{calc}g/cm^3	1.703
μ/mm^{-1}	3.646
F(000)	1680.0
Crystal size/mm^3	0.160 × 0.150 × 0.140
Radiation	CuKα (λ = 1.54178)
2Θ range for data collection/°	8.028 to 132.018
Index ranges	-8 ≤ h ≤ 13, -19 ≤ k ≤ 26, -18 ≤ l ≤ 14
Reflections collected	10063
Independent reflections	5641 [R_{int} = 0.0459, R_{sigma} = 0.0651]
Data/restraints/parameters	5641/0/487
Goodness-of-fit on F^2	1.005
Final R indexes [I>=2σ (I)]	R_1 = 0.0494, wR_2 = 0.1180
Final R indexes [all data]	R_1 = 0.0616, wR_2 = 0.1280
Largest diff. peak/hole / e Å^{-3}	0.88/-0.76

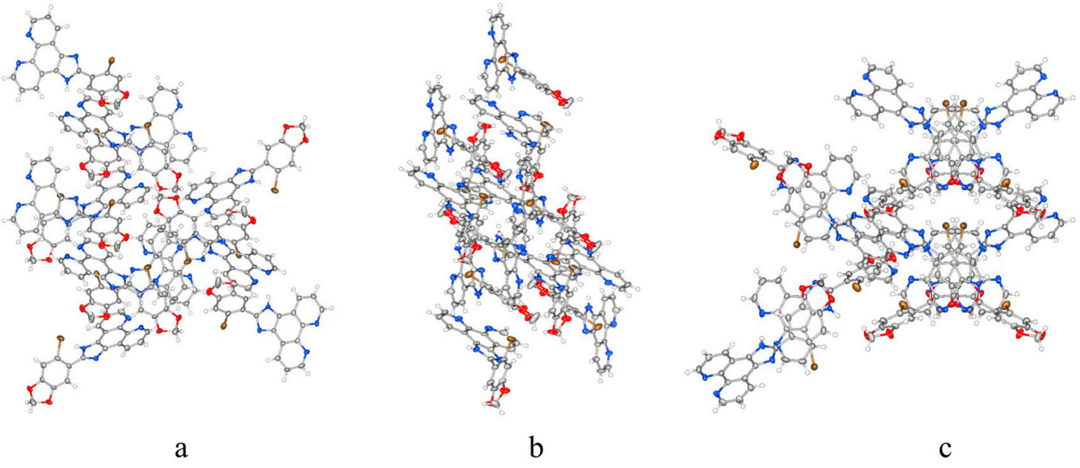

Fig. S1. Molecular packing of compound 5 viewed along the a, b, and c crystallographic axes.

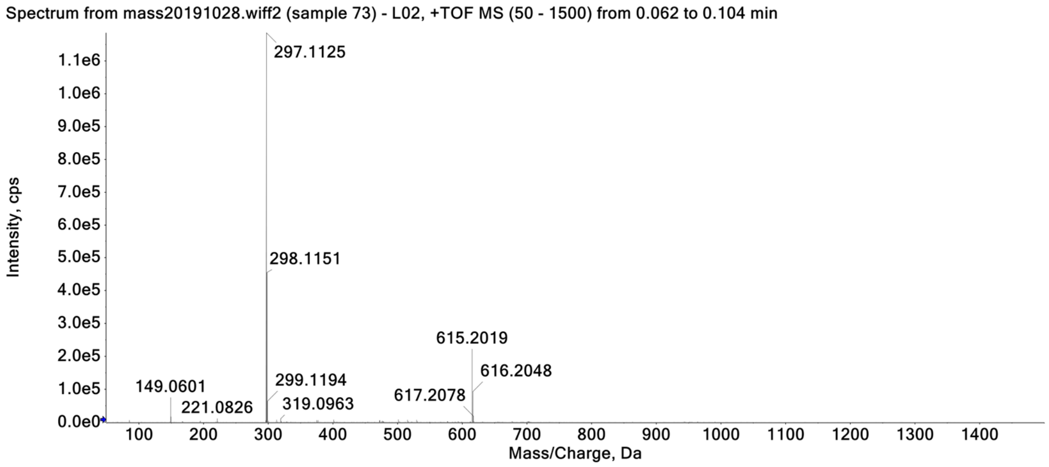

Fig. S2. ESI-MS spectrum of 1.

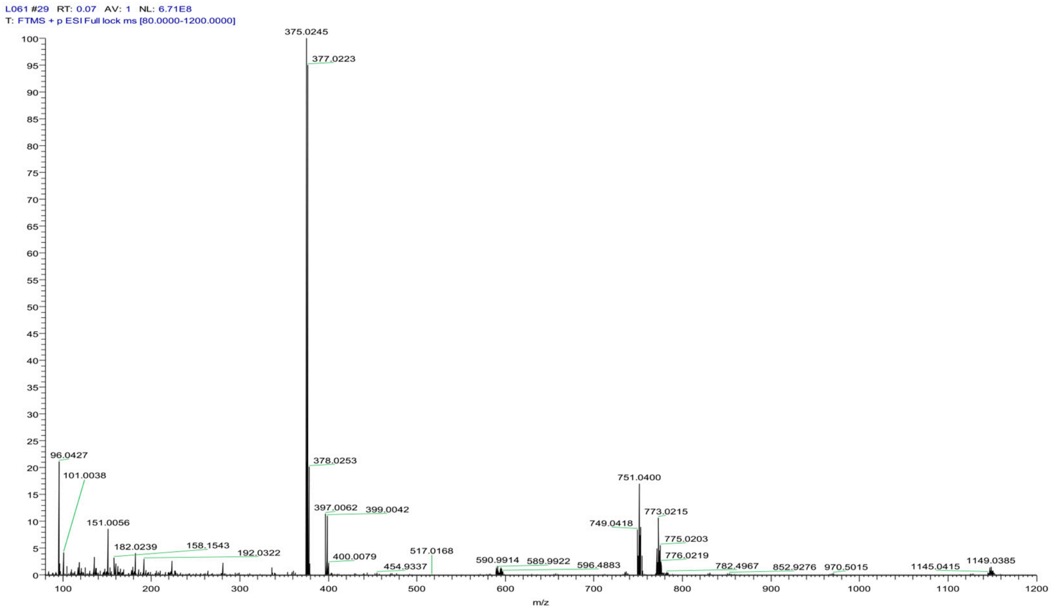

Fig. S3. ESI-MS spectrum of 2.

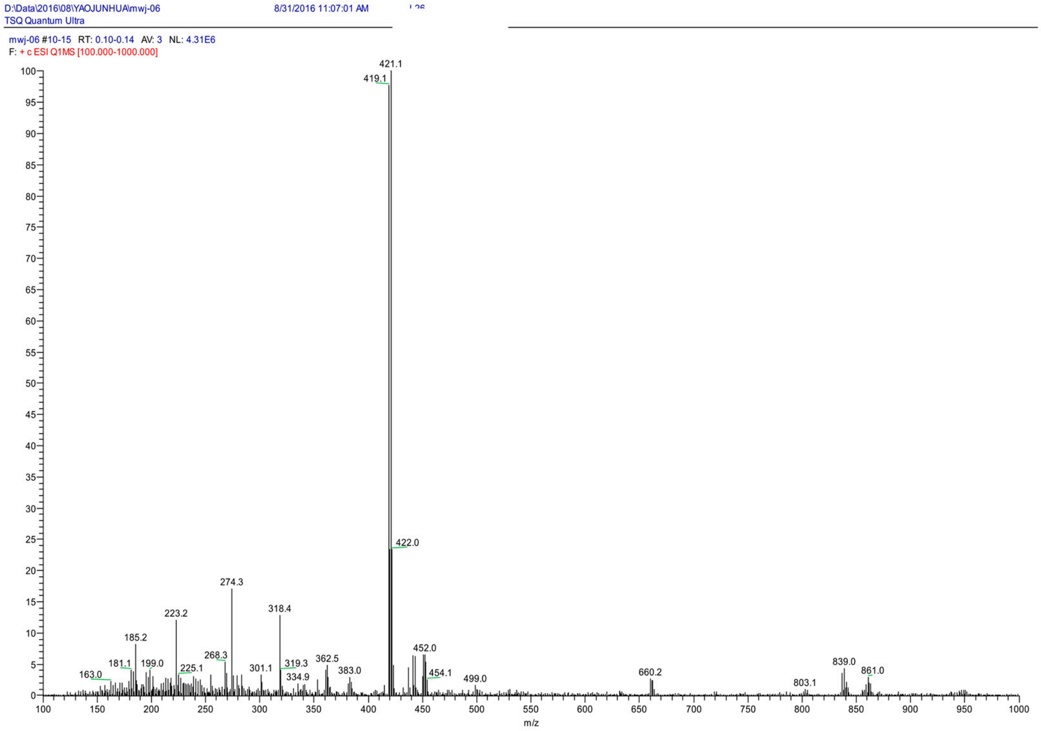

Fig. S4. ESI-MS spectrum of 3.

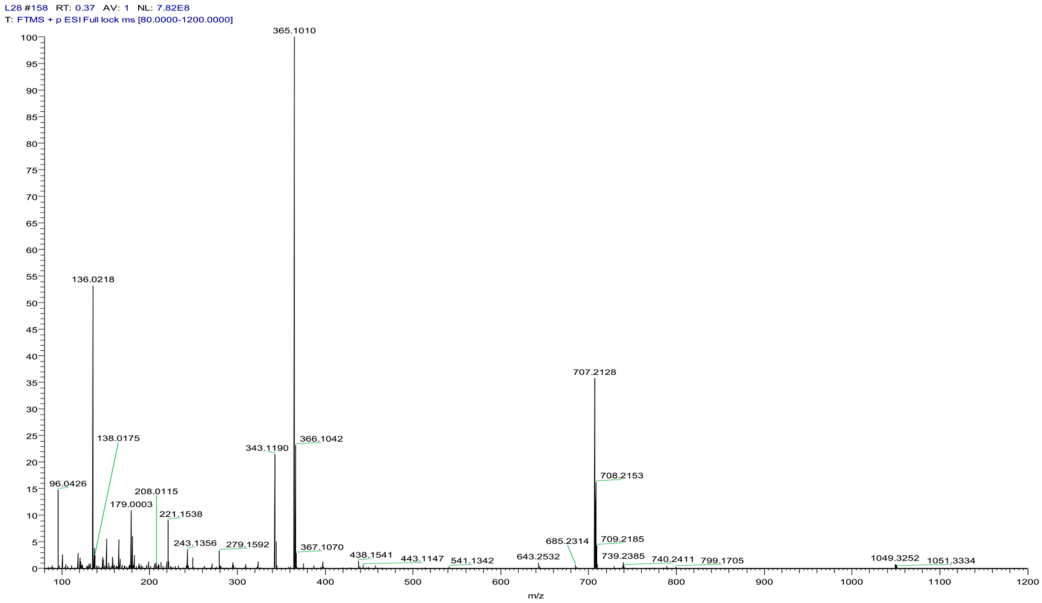

Fig. S5. ESI-MS spectrum of 4.

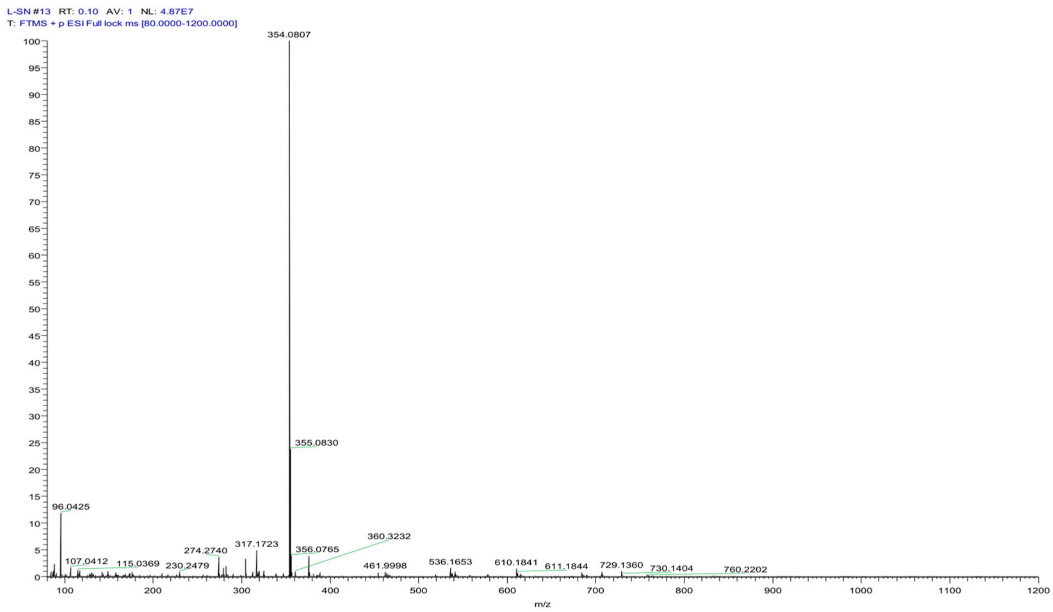

Fig. S6. ESI-MS spectrum of 5.

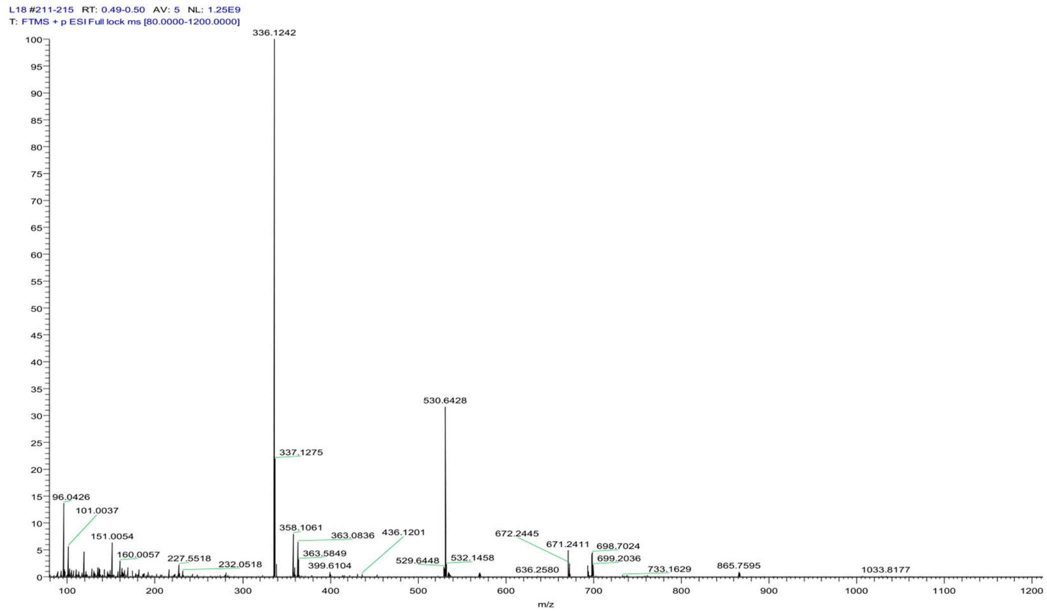

Fig. S7. ESI-MS spectrum of 6.

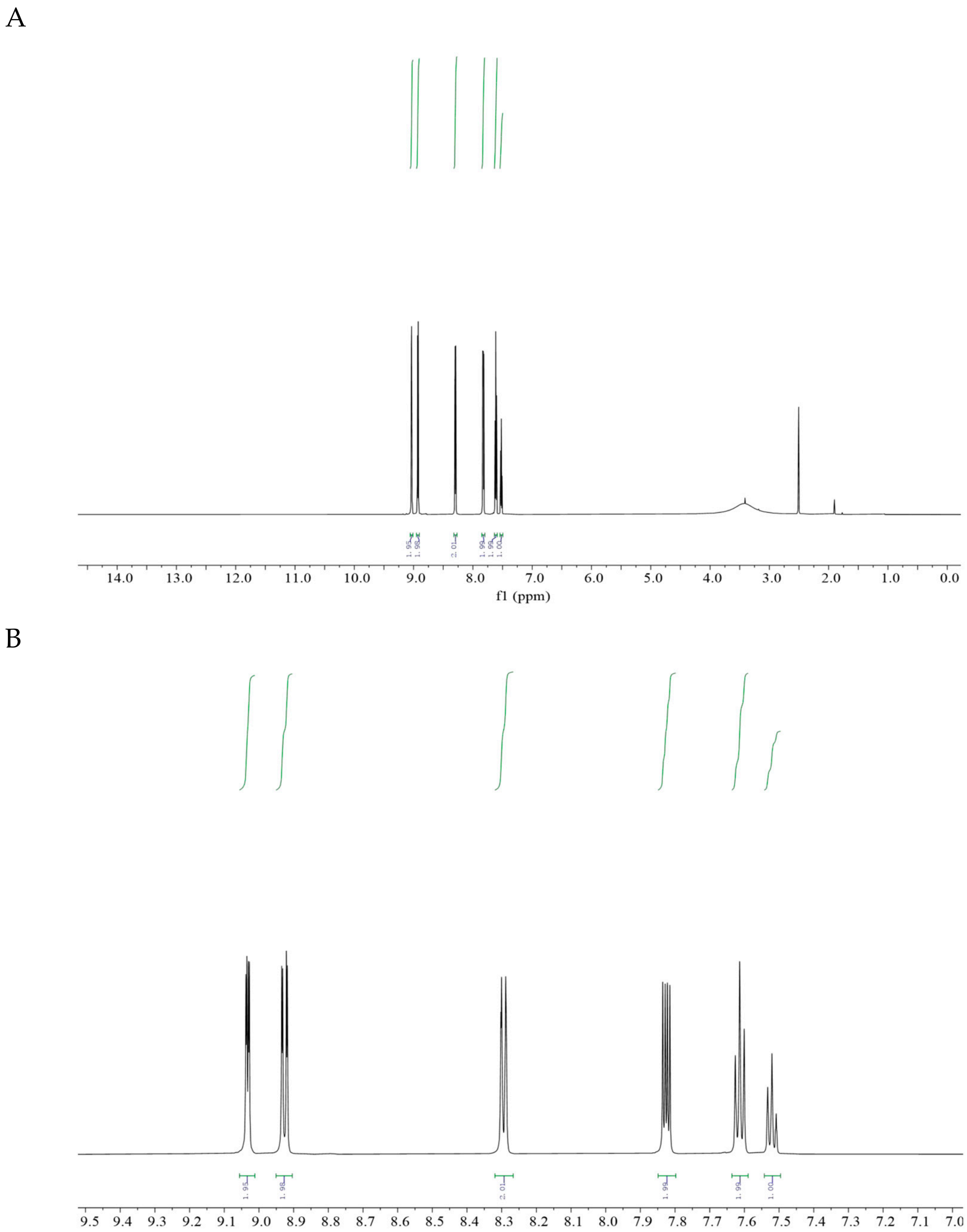

Fig. S8. The whole version (A) and enlarge version (B) of ^{1}H NMR spectrum of 1.

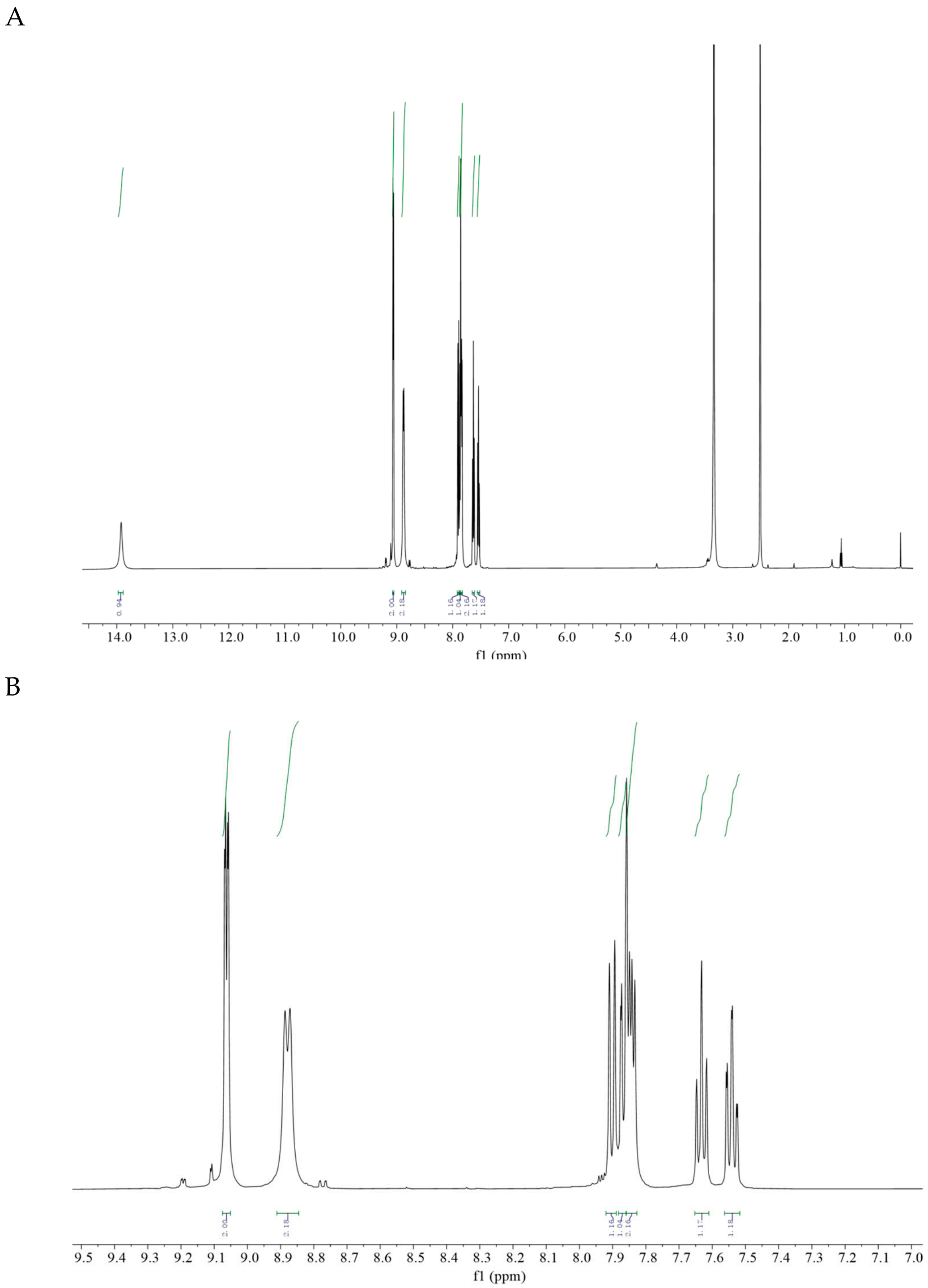

Fig. S9. The whole version (A) and enlarge version (B) of ^{1}H NMR spectrum of 2.

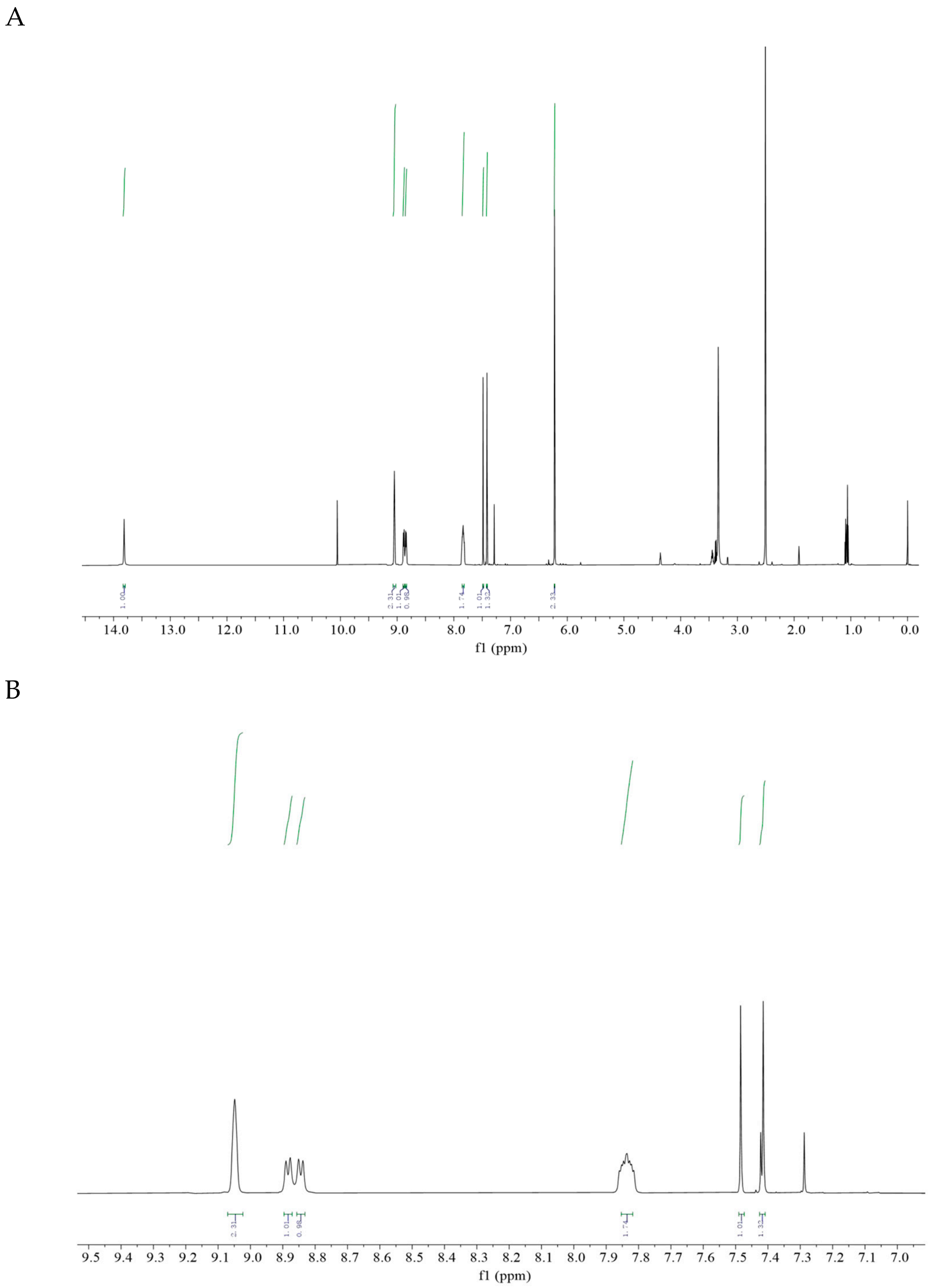

Fig. S10. The whole version (A) and enlarge version (B) of ^{1}H NMR spectrum of 3.

A

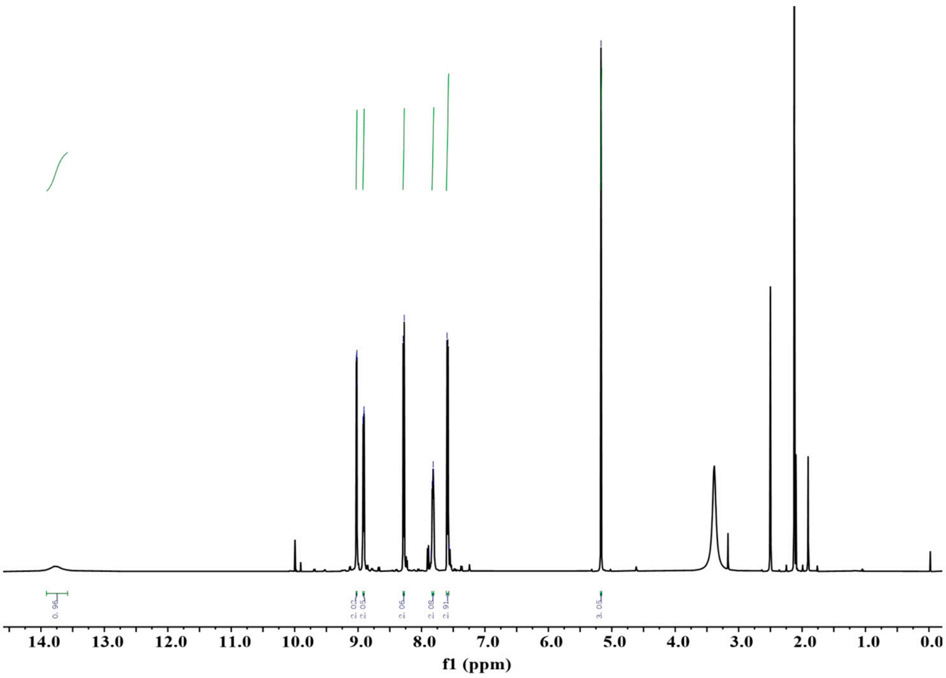

B

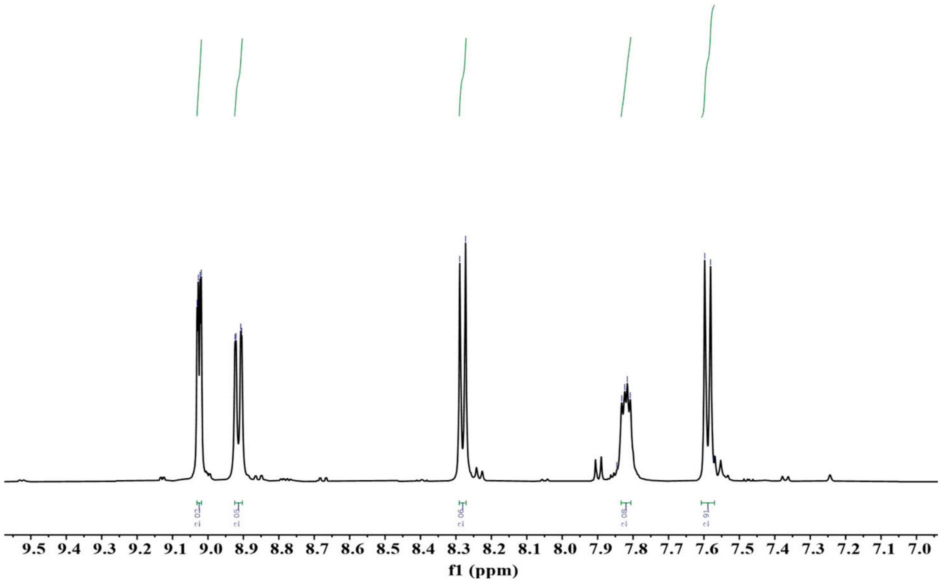

Fig. S11. The whole version (A) and enlarge version (B) of ^{1}H NMR spectrum of 4.

A

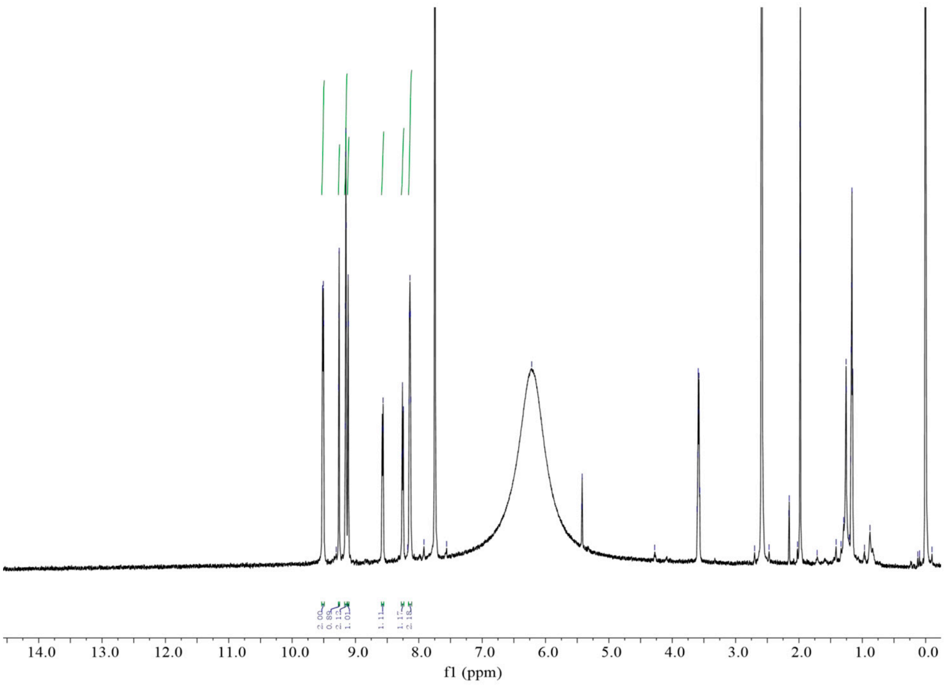

B

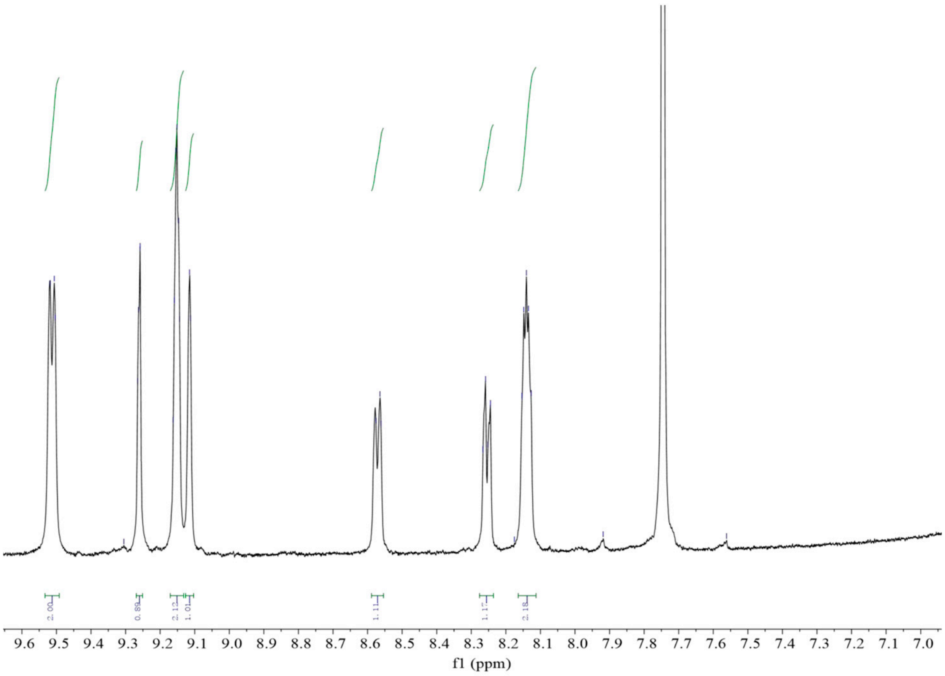

Fig. S12. The whole version (A) and enlarge version (B) of ^{1}H NMR spectrum of 5.

A

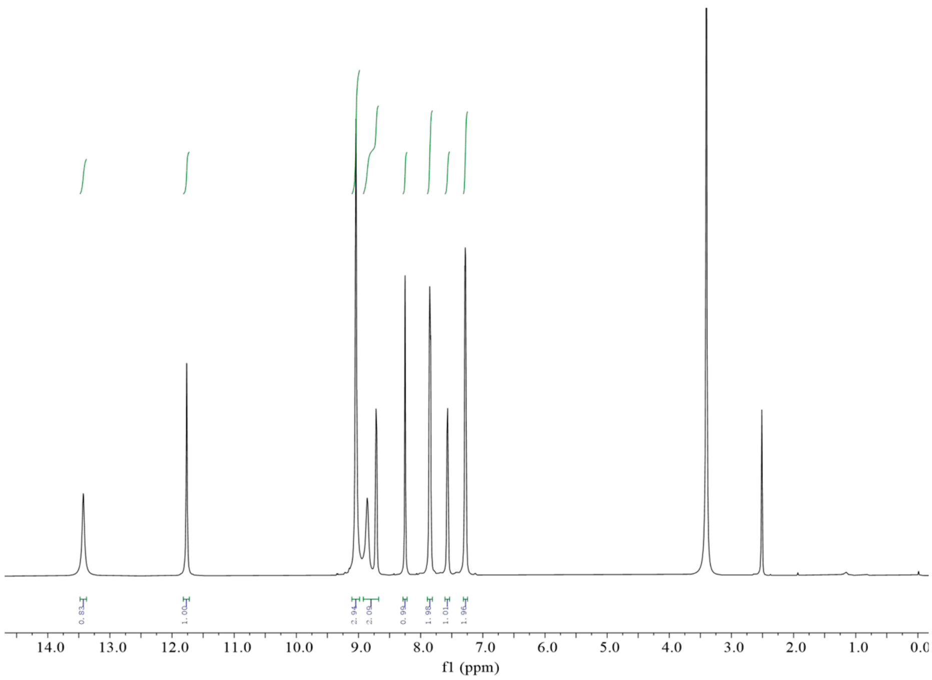

B

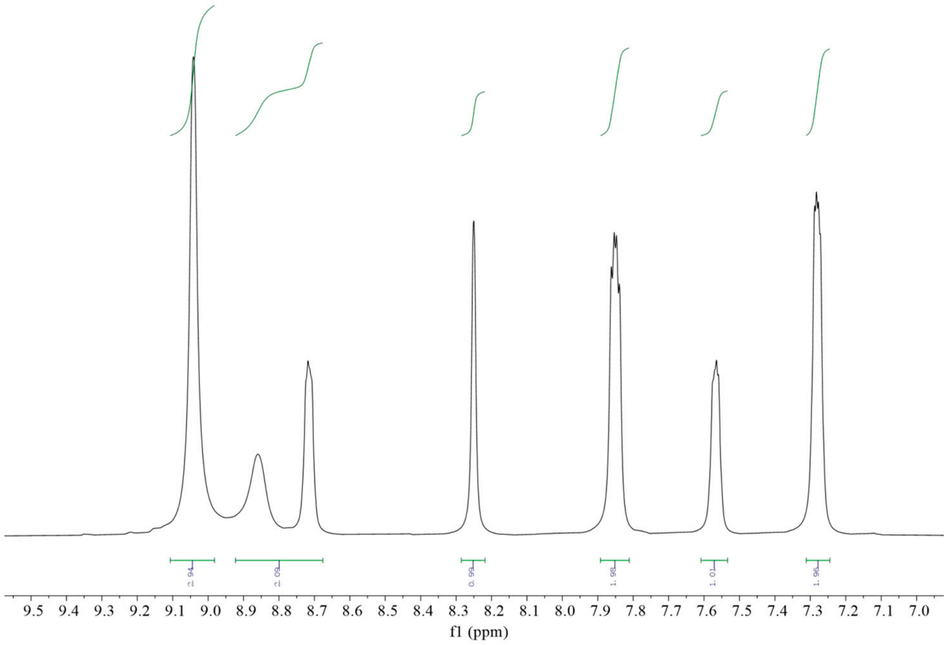

Fig. S13. The whole version (A) and enlarge version (B) of ^{1}H NMR spectrum of 6.

A

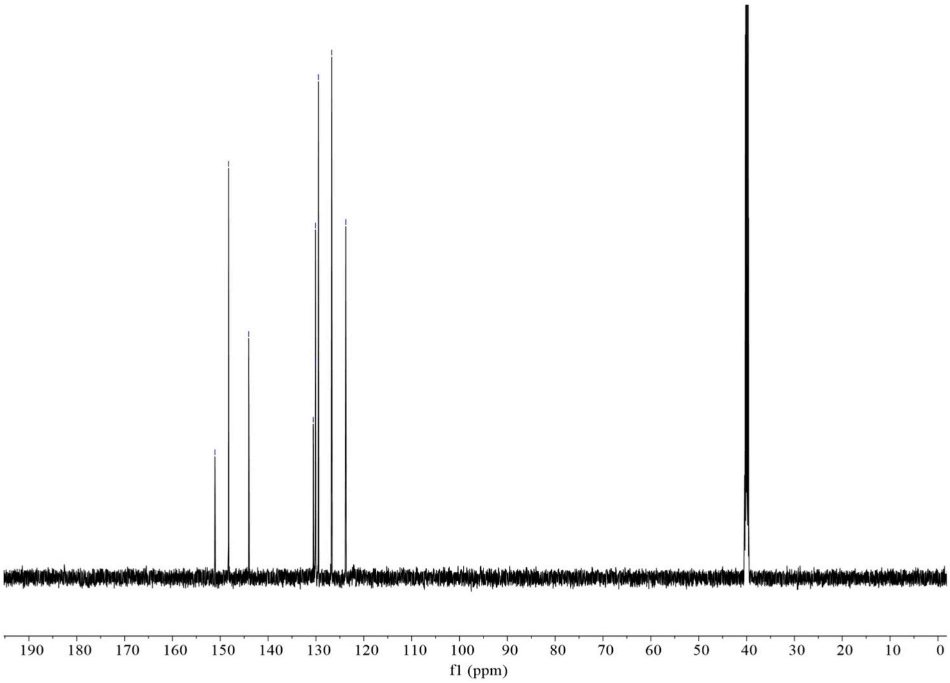

B

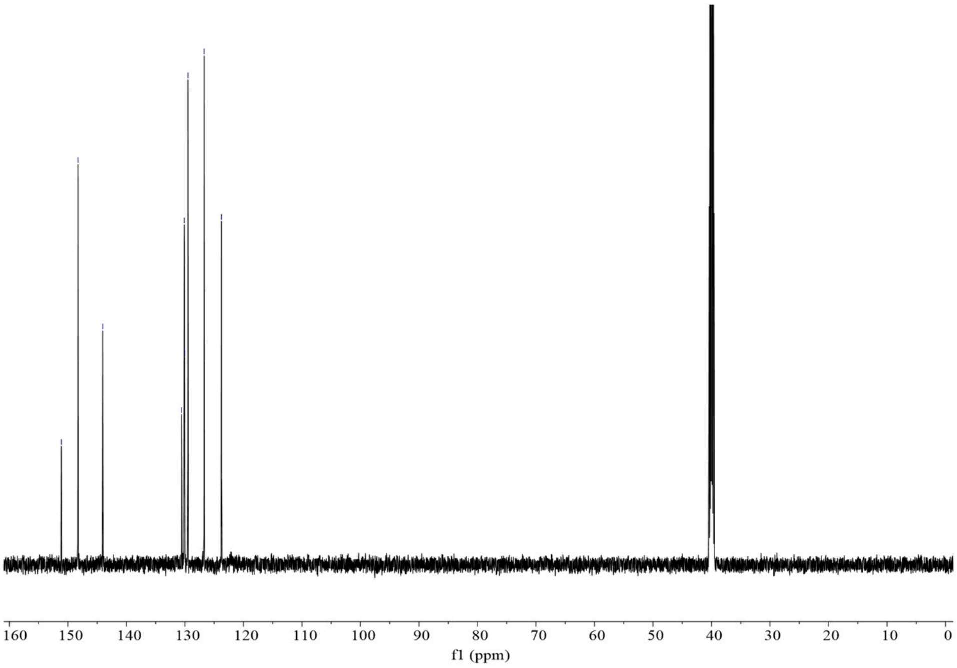

Fig. S14. The whole version (A) and enlarge version (B) of ^{13}C NMR spectrum of 1.

A

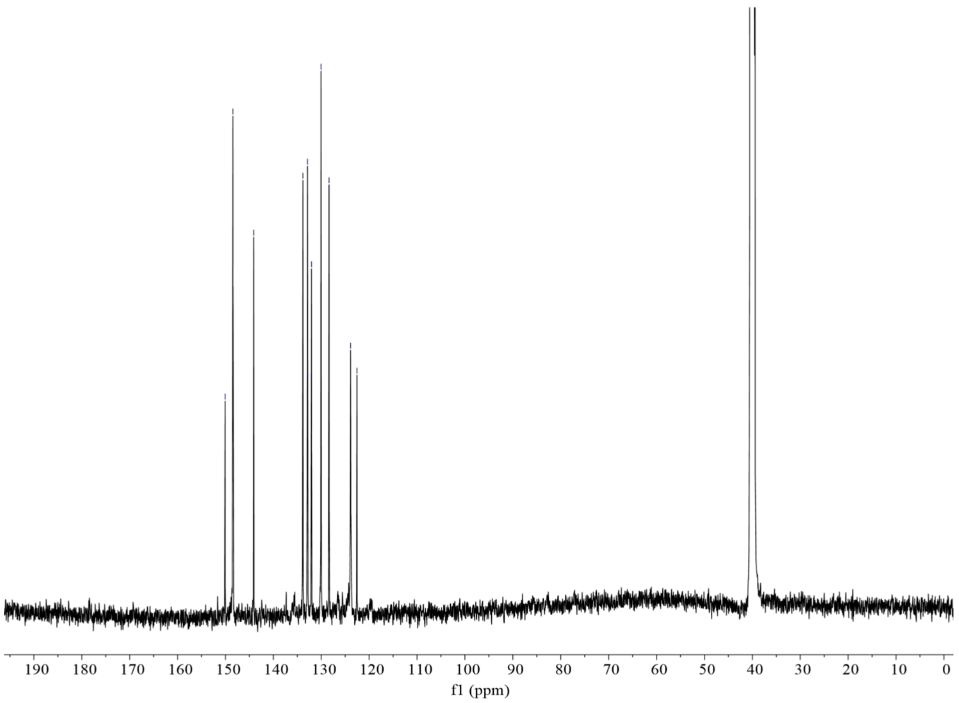

B

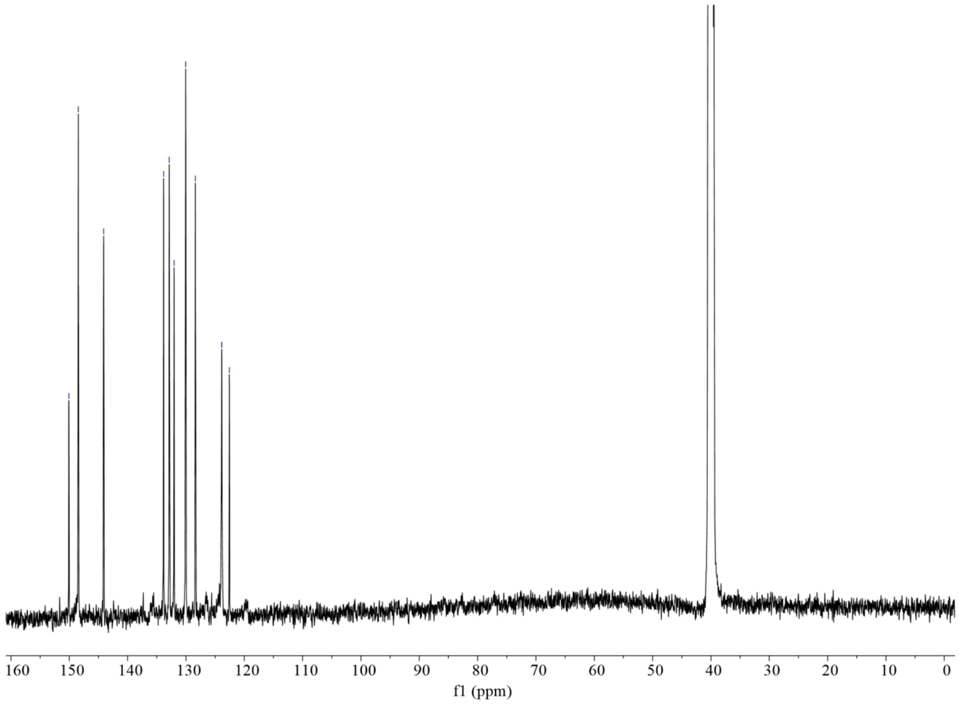

Fig. S15. The whole version (A) and enlarge version (B) of ^{13}C NMR spectrum of 2.

A

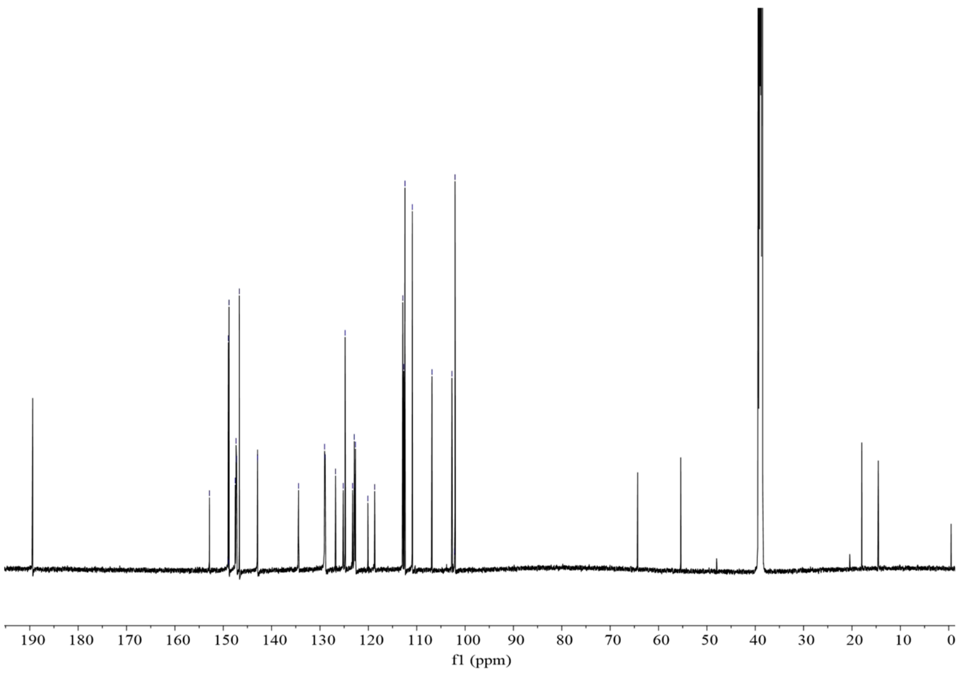

B

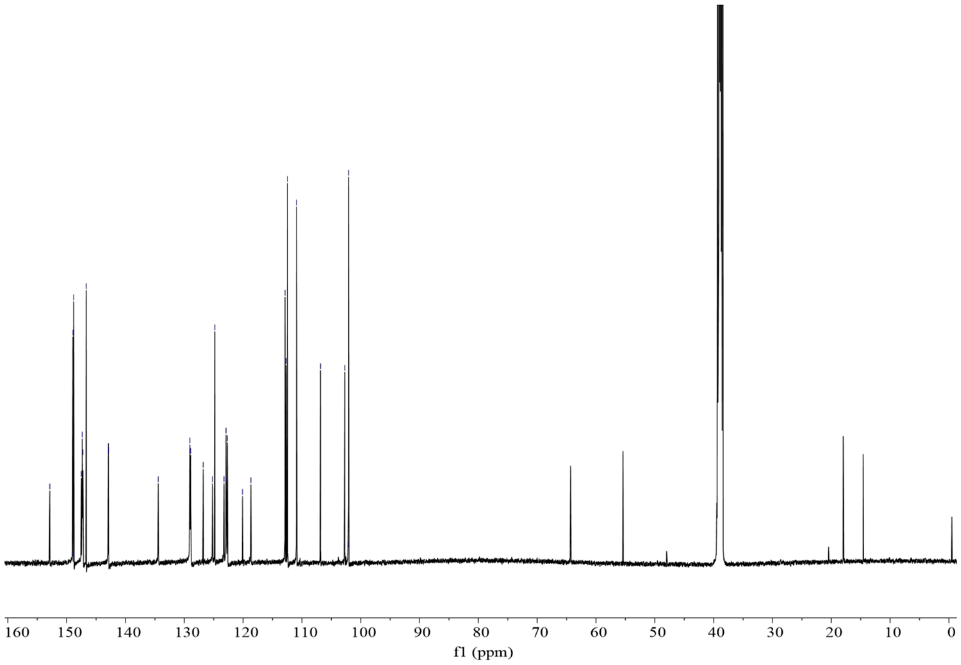

Fig. S16. The whole version (A) and enlarge version (B) of ^{13}C NMR spectrum of 3.

A

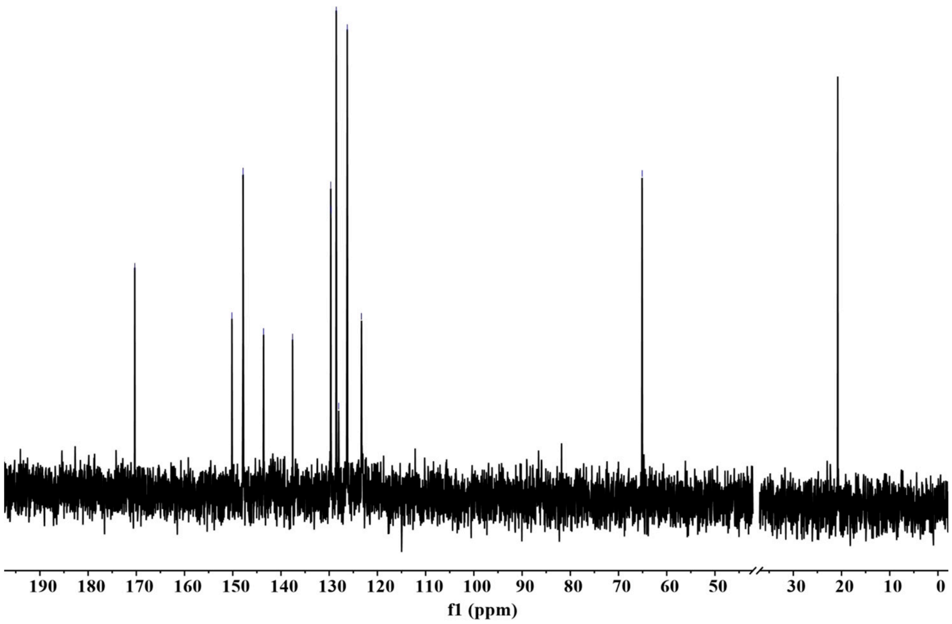

B

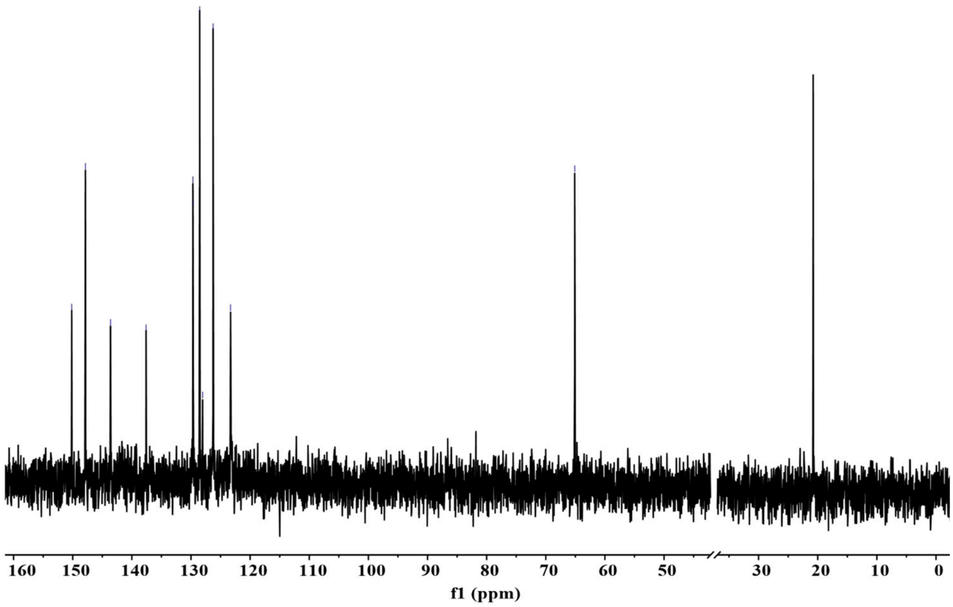

Fig. S17. The whole version (A) and enlarge version (B) of ^{13}C NMR spectrum of 4.

A

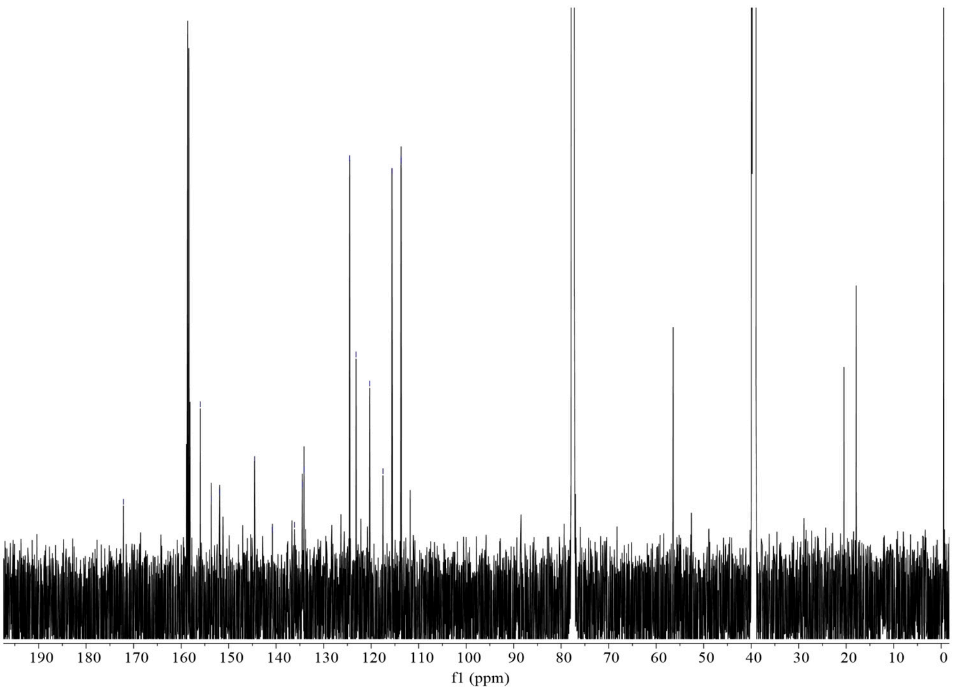

B

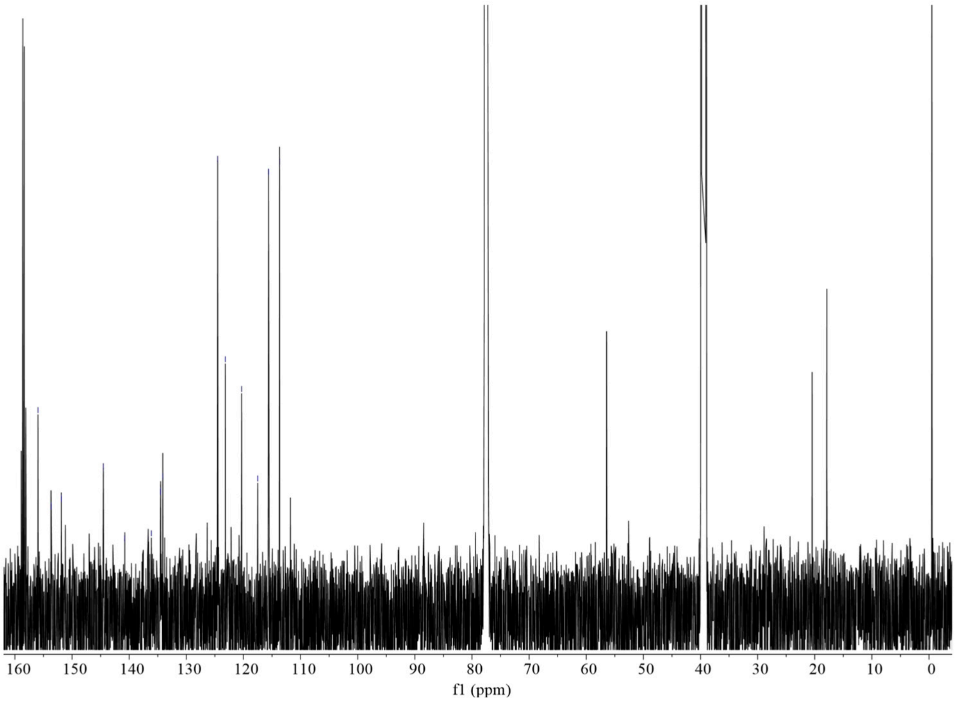

Fig. S18. The whole version (A) and enlarge version (B) of ^{13}C NMR spectrum of 5.

A

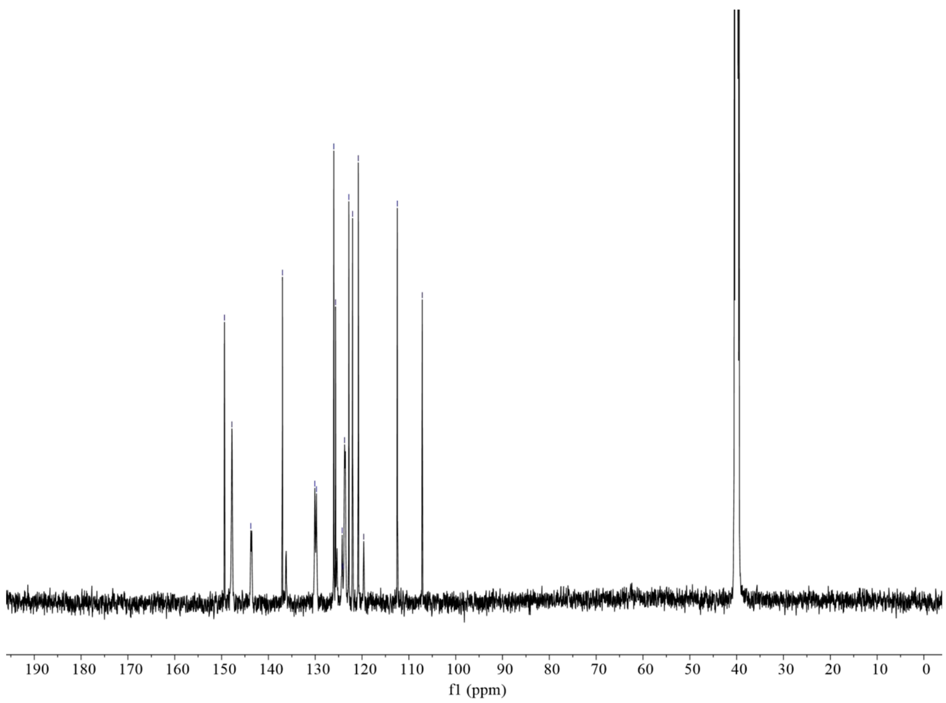

B

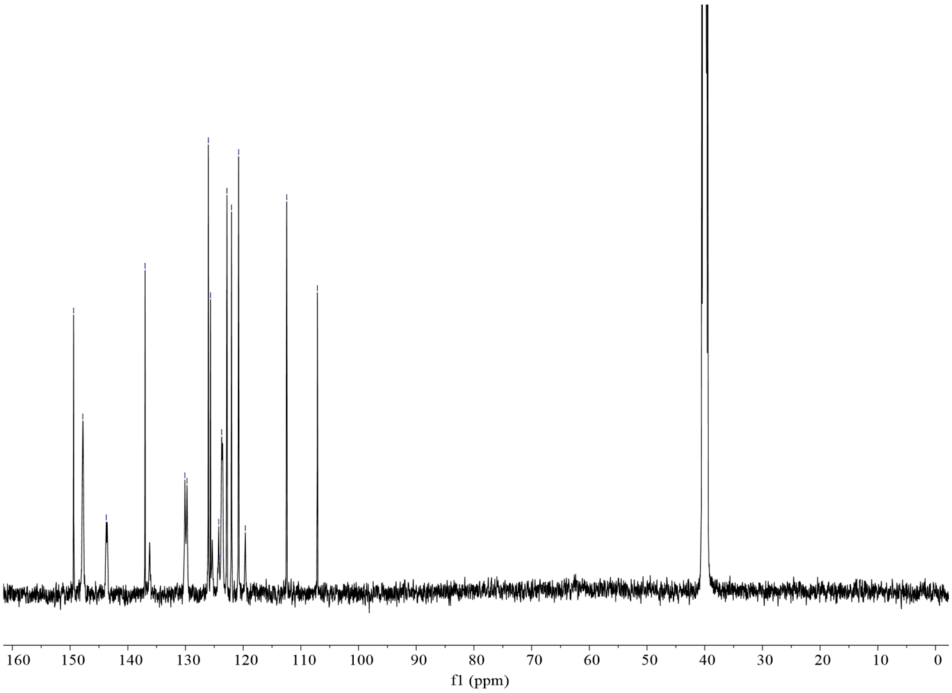

Fig. S19. The whole version (A) and enlarge version (B) of ^{13}C NMR spectrum of 6.

Table S3. ITC binding isotherm of 3 (10 μM) titrated into EGFR (10 μM) or KRAS (10 μM) in Tris-HCl (pH 7.4, 298 K).

DNA	**$K_d(M^{-1})$**	**$\Delta G(kJ\cdot mol^{-1})$**	**$\Delta H(kJ\cdot mol^{-1})$**	**$T\Delta S(kJ\cdot mol^{-1})$**	**n**
KRAS	1.49×10^{-5}	-27.54	-95.43	-67.85	7.598

EGFR	1.02×10^{-7}	-39.88	-91.60	-51.73	0.33

Table S4 Acid dissociation constant (pKa) data for 1 in 20% (v/v) ethanol-water solution

Compound 1				
**pH**	**A**	**lg(A-A_{In-})/(A_{HIn}-A)**	**pKa**	**Mean**
5.79	1.313	0.28	6.07	
5.61	1.326	0.33	5.94	
5.41	1.322	0.31	5.72	
5.20	1.339	0.38	5.58	5.48
5.00	1.317	0.29	5.29	
4.81	1.280	0.16	4.97	
4.59	1.289	0.19	4.78	

A_{In-}=0.987，A_{HIn}=1.485，λ=290 nm，25±0.5℃

Table S5 Acid dissociation constant (pKa) data for 2 in 20% (v/v) ethanol-water solution

Compound 2				
**pH**	**A**	**lg(A-A_{In-})/(A_{HIn}-A)**	**pKa**	**Mean**
5.79	0.893	0.29	6.08	
5.61	0.900	0.34	5.95	
5.41	0.908	0.41	5.82	
5.20	0.909	0.41	5.61	5.60
5.00	0.917	0.48	5.48	
4.81	0.904	0.37	5.18	
4.59	0.916	0.47	5.06	

A_{In-}=0.725，A_{HIn}=0.980，λ=290 nm，25±0.5℃

Table S6 Acid dissociation constant (pKa) data for 3 in 20% (v/v) ethanol-water solution

Compound 3				
pH	A	$lg(A-A_{In-})/(A_{HIn}-A)$	pKa	Mean
5.79	0.796	-0.18	5.61	
5.61	0.765	-0.66	4.95	
5.41	0.757	-0.85	4.56	
5.20	0.782	-0.37	4.83	4.83
5.00	0.808	-0.04	4.96	
4.81	0.803	-0.10	4.71	
4.59	0.781	-0.39	4.20	

A_{In-}=0.739，A_{HIn}=0.883，λ=290 nm，25±0.5℃

Table S7 Acid dissociation constant (pKa) data for 4 in 20% (v/v) ethanol-water solution

Compound 4				
pH	A	$lg(A-A_{In-})/(A_{HIn}-A)$	pKa	Mean
5.79	0.569	-0.11	5.68	
5.61	0.575	-0.05	5.56	
5.41	0.577	-0.03	5.38	
5.20	0.573	-0.07	5.13	5.16
5.00	0.580	0.01	5.01	
4.81	0.582	0.03	4.84	
4.59	0.575	-0.05	4.54	

A_{In-}=0.495，A_{HIn}=0.664，λ=295 nm，25±0.5℃

Table S8 Acid dissociation constant (pKa) data for 6 in 20% (v/v) ethanol-water solution

Compound 6				
**pH**	**A**	**lg(A-A_{In-})/(A_{HIn}-A)**	**pKa**	**Mean**
5.79	1.006	-0.50	5.29	
5.61	1.010	-0.53	5.08	
5.41	1.011	-0.53	4.88	
5.20	1.016	-0.56	4.64	4.82
5.00	0.962	-0.27	4.73	
4.81	0.945	-0.19	4.62	
4.59	0.918	-0.07	4.52	

A_{In-}=1.101，A_{HIn}=0.705，λ=285 nm，25±0.5℃

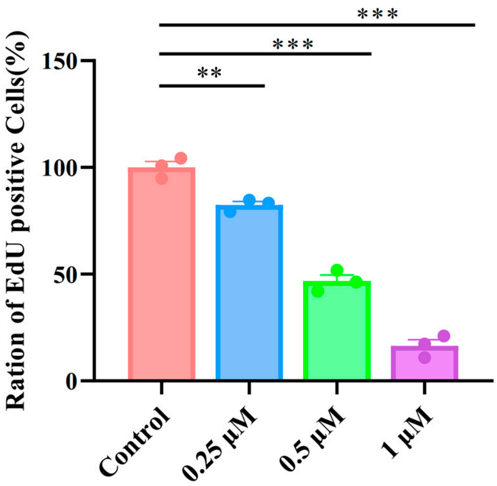

Fig. S20. Quantitative analysis of EdU positive cell ratio normalized to control.

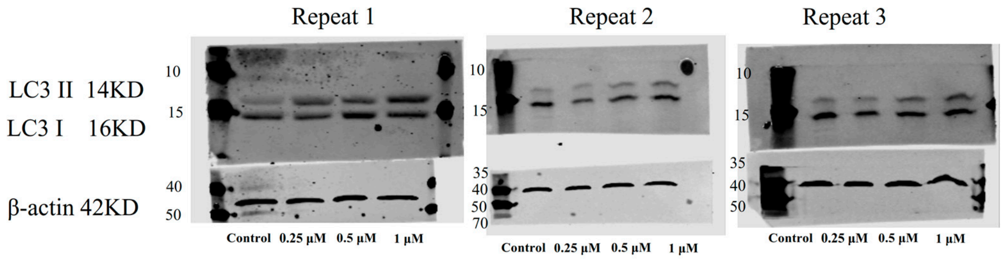

Fig. S21. LC3B of original Western blot for three repeats.

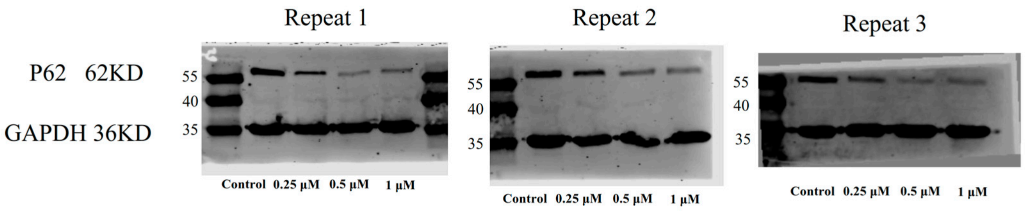

Fig. S22. p62 of original Western blot for three repeats.

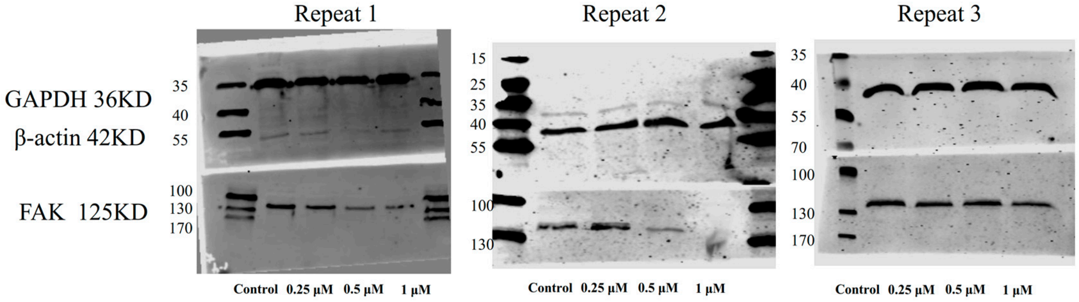

Fig. S23. FAK of original Western blot for three repeats.

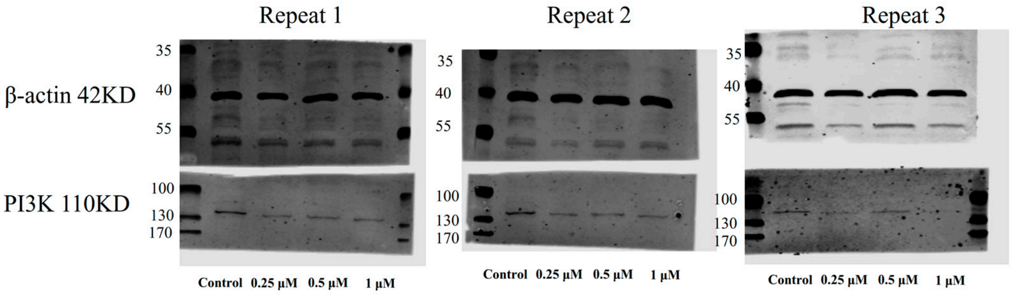

Fig. S24. PI3K of original Western blot for three repeats.

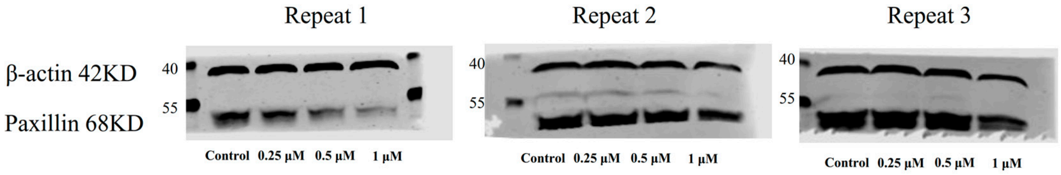

Fig. S25. Paxillin of original Western blot for three repeats.

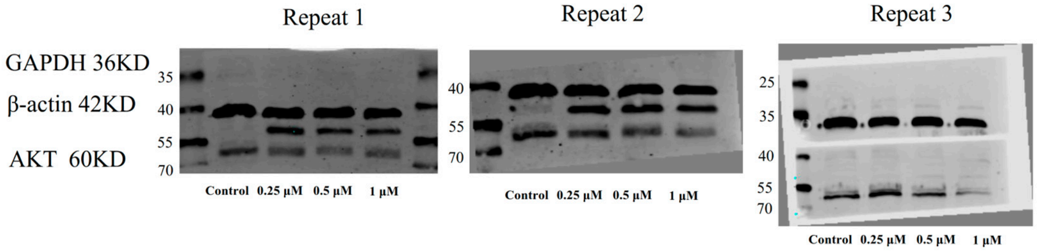

Fig. S26. AKT of original Western blot for three repeats.

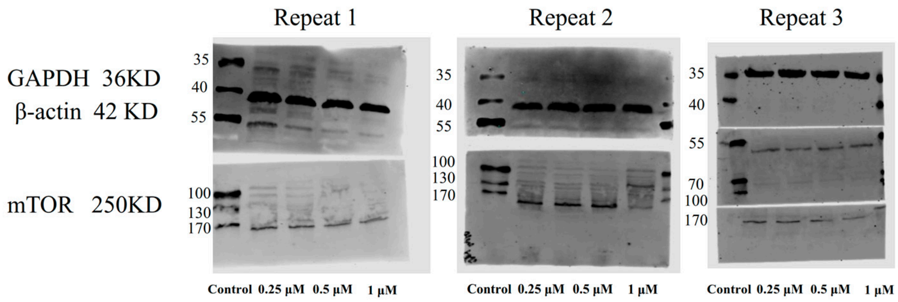

Fig. S27. mTOR of original Western blot for three repeats.

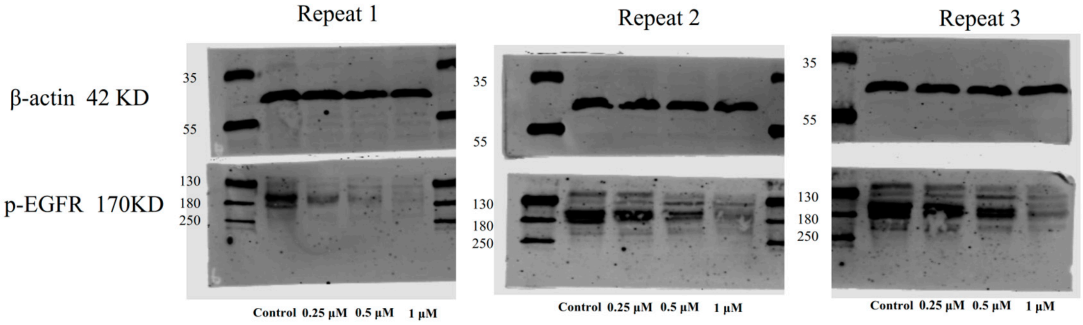

Fig. S28. p-EGFR of original Western blot for three repeats.

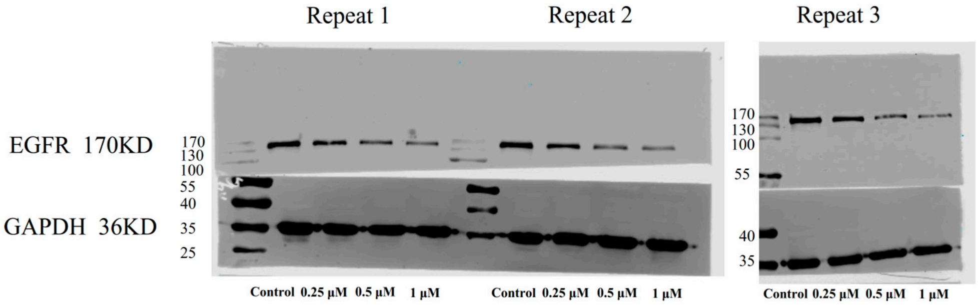

Fig. S29. EGFR of original Western blot for three repeats.

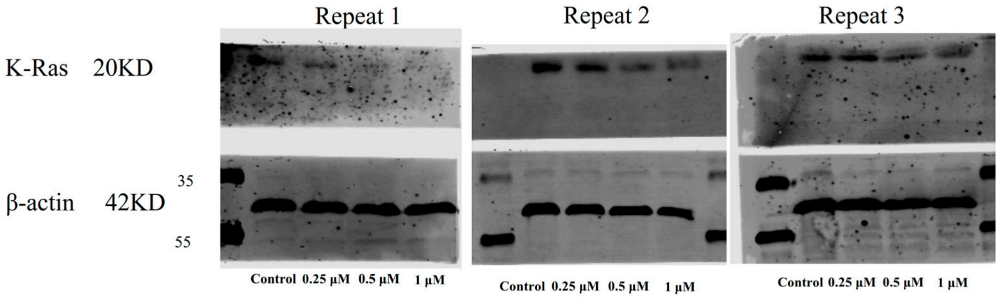

Fig. S30. K-Ras of original Western blot for three repeats.

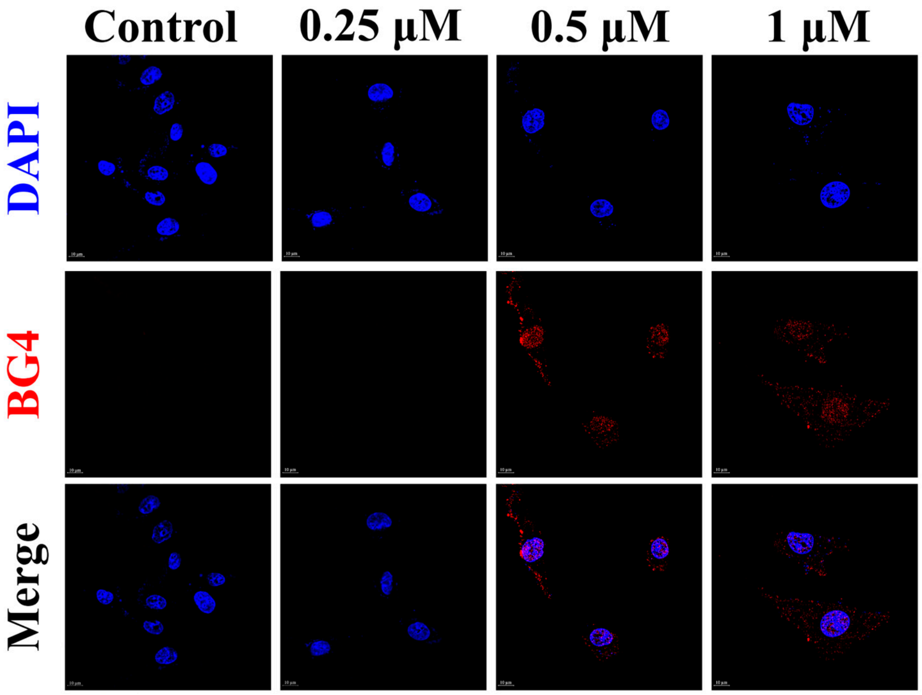

Fig. S31. Immunofluorescence staining of BG4 (red) with DAPI (blue) to evaluate G-quadruplex (G4) stabilization in U87-MG cells treated with compound 3.

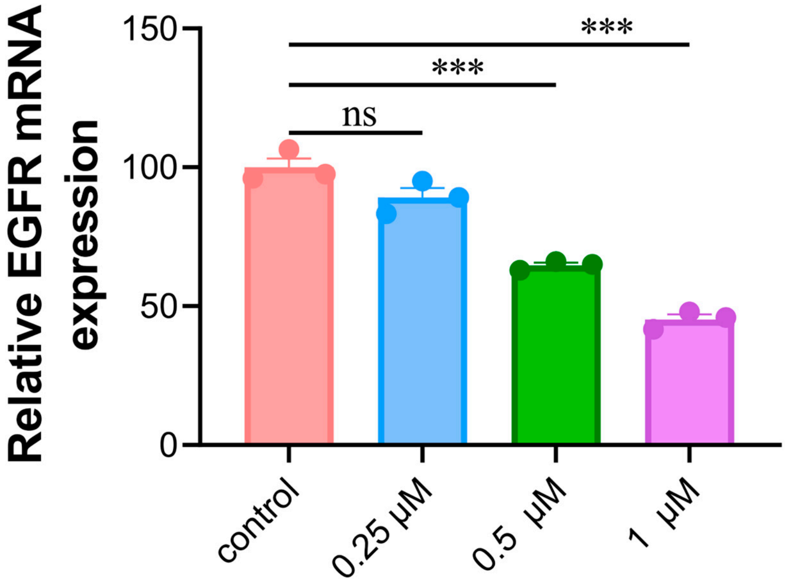

Fig. S32. Relative EGFR mRNA expression levels in U87-MG cells treated with compound 3 (0, 0.25, 0.5, 1 μM) for 72 h, measured by quantitative real-time PCR. Data are presented as mean ± SEM (n = 3). *$p < 0.05$, **$p < 0.01$, ***$p < 0.001$ vs. control group.
